# The type of food influences the behaviour of *Listeria monocytogenes* in a food-gastrointestinal-infection model

**DOI:** 10.1038/s41538-025-00436-5

**Published:** 2025-05-19

**Authors:** Nadja Pracser, Andreas Zaiser, Luminita Ciolacu, Franz-Ferdinand Roch, Narciso M. Quijada, Sarah Thalguter, Monika Dzieciol, Beate Conrady, Martin Wagner, Kathrin Rychli

**Affiliations:** 1 Centre for Food Science and Veterinary Public Health, Clinical Department for Farm Animals and Food System Science, University of Veterinary Medicine Vienna, Vienna, Austria; 2 FFoQSI GmbH-Austrian Competence Centre for Feed and Food Quality, Safety and Innovation, Tulln, Austria; 3 Department of Microbiology and Genetics, Institute for Agribiotechnology Research (CIALE), University of Salamanca, Salamanca, Spain; 4 Department of Veterinary and Animal Sciences, University of Copenhagen, Frederiksberg C, Copenhagen, Denmark

**Keywords:** Bacteria, Pathogens

## Abstract

Food contaminated with *Listeria (L.) monocytogenes* is the main source of human listeriosis, but how different food matrices affect the survival and invasion in the gastrointestinal (GI) tract is still unclear. This study examined three ready-to-eat foods - soft-cheese, smoked salmon, and sausage - using a food-GI-infection model. We observed strain-dependent growth rates, but food matrices did not significantly impact growth. However, nutrient sources altered gene expression. Passage through the GI model upregulated 23 stress genes and 29 virulence genes (e.g., *clpE*, *hly*, and *plcB*). *L. monocytogenes* survival was higher in cheese and fish compared to sausage, due to their lower buffer capacity. Invasion efficiency into Caco-2 cells was highest in fish, potentially linked to its fatty acid composition. Food matrices and GI conditions influenced the transcriptional profiles of stress-associated and virulence genes. This study highlights the significant role of food matrices in *L. monocytogenes* survival and infection.

## Introduction

Listeriosis, a rare but severe disease with high mortality rates, is induced by the Gram-positive bacterium *Listeria* (*L*.) *monocytogenes*^1^. In the vast majority of listeriosis cases, contaminated food is the infection source^2^. In healthy individuals, the major symptom of listeriosis defines as a non-invasive, self-limiting gastroenteritis; however, in immunocompromised and elderly individuals, a severe and systemic infection can occur, resulting in meningoencephalitis or septicaemia^1^. Listeriosis outbreaks and sporadic cases have been reported worldwide^3^ and were linked to contaminated fish and fishery products, meat and meat products, cheeses, and vegetables. E.g., there was a notable multinational outbreak linked to the consumption of cold-smoked fish products from 2014 to 2019^4^. In 2009/2010, a listeriosis outbreak occurred in Austria, Germany, and the Czech Republic^5^, which was associated with contaminated “Quargel” cheese, a type of acid curd cheese. Furthermore, in 2021, a soft cheese was the source of an outbreak in the US^6^. The outbreak in South Africa, linked to ready-to-eat (RTE) sausage, was declared the world’s largest listeriosis outbreak with 1060 confirmed cases, including 216 deaths^7^.

*L. monocytogenes* can be found in raw products and processed foods that are contaminated during and/or after processing. As *L. monocytogenes* can multiply at low temperatures (2 to 4 °C), RTE foods with relatively long shelf lives (such as fishery products, meat products, and cheese) are of particular public health concern^2^. Data of RTE food samples provided by EU member states indicate a *L. monocytogenes* occurrence of 7.1% in fish and fishery products, 2.1% in RTE meat and meat products and 2% in soft and semi-soft cheeses^3^. Food contamination with *L. monocytogenes* results in millions of euros of economic loss in Europe and the US each year due to product recalls, reduced productivity, and increased medical care costs. In 2012, the total annual cost of listeriosis was calculated to be 2.6 billion dollars in the US alone^8^.

In the human gastrointestinal (GI) tract, *L. monocytogenes* needs to withstand low pH levels and bile stress^9^. Research suggests that the composition of food or specific food components impact the survival of *Listeria* during exposure to gastric juices^10–14^. Moreover, studies showed that the food matrices and/or food components modulate expression of virulence genes and virulence in vitro and in vivo^15–22^. However, the results of these studies were contradictory.

The main drawback of all the previous studies is that they focused only on a single part of the natural route of *L. monocytogenes* infection (either survival in the GI tract or infection) with a single exception: Colás-Medà et al. reported changes in the invasion ability of *L. monocytogenes* growing on pears and melons and surpassing a three-steps digestion model^23^. Therefore, current knowledge about the effect of food matrices, especially of epidemiologically relevant RTE food, on survival in the GI tract and subsequent virulence of *L. monocytogenes* is very limited.

To address this research question, we implemented a complex food-GI-infection model mimicking the route of infection during human listeriosis. The model included the growth of *L. monocytogenes* in RTE food at low temperature storage conditions, a three-steps in vitro digestive model (consisting of an oral, a gastric, and an intestinal phase), and an in vitro virulence assay using human intestinal epithelial Caco-2 cells. We selected three epidemiologically relevant food matrices (“Brie” soft cheese, smoked salmon, and heat-treated non-fermented sausage (knackwurst)) representing high-risk RTE food categories, which are frequently involved in foodborne listeriosis outbreaks^3^. Our objective was to explore if the growth of *L. monocytogenes*, the survival in an artificial digestive model, and the virulence were influenced by the food type. We further investigated the gene expression profile of *L. monocytogenes* in the different food matrices and at the end of the GI model.

## Results

### Growth of *L. monocytogenes* in different food matrices

**Fig. 1:**
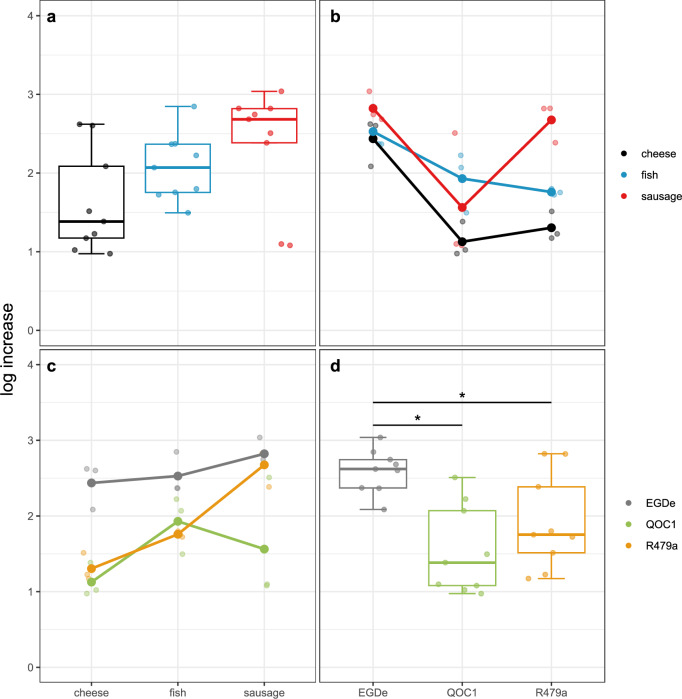
Growth of *L. monocytogenes* in different food matrices. Boxplot for log increase split into the different food matrices (**a**) and the different strains (**d**). Interaction plot for log increase showing interactions of the different food matrix depending on the strain (**b**) and of the different strains depending on the food matrix (**c**). Black: cheese (*n* = 9), blue: fish (*n* = 9), red: sausage (*n* = 9), grey: EGDe (*n* = 9), green: QOC1 (*n* = 9), orange: R479a (*n* = 9). *p < 0.05.

Several interaction effects were observed between the food matrices and the three *L. monocytogenes* strains (Fig. 1). While the type of food did not significantly affect bacterial growth after seven days at 10 °C, notable differences were seen among the strains. Specifically, strain EGDe showed significantly higher growth in all tested food matrices (mean log increase = 2.600) compared to the QOC1 strain (mean log increase = 1.540, *p*-value 0.0007) and the R479a strain (mean log increase = 1.913, *p*-value 0.0007; Fig. 1d and Table S3 for details). The pH levels of the three food matrices were similar, with 5.91 for cheese, 5.94 for fish, and 5.81 for sausage (Fig. S1).

### Survival of *L. monocytogenes* after oral, gastric, and intestinal phases

We analysed the survival of the strains EGDe, QOC1, and R479a, which were cultivated for seven days at 10 °C in cheese, fish, and sausage in the GI model after the oral, gastric, and intestinal phases. Notably, we observed multiple interactions between *L. monocytogenes* strains and food matrices. The detected interactions, particularly after the gastric and intestinal phases, can be primarily attributed to the unique properties of the R479a strain in cheese.

Upon comparing the log reduction after the oral phase, we observed significant differences both between the strains (*p*-value = 0.0040) and the food matrices (*p*-value = 0.041, Fig. S2 and Table S1). Notably, the R479a strain exhibited significantly higher survival rates compared to the EGDe (*p*-value = 0.0159) and QOC1 strains (*p*-value = 0.0059). Interestingly, bacteria preincubated in sausage survived better during saliva digestion compared to those in cheese, although the difference was minor. The pH levels post-saliva digestion (pH 7.46) were similar across the food matrices (pH of 6.02 for cheese, 6.16 for fish, and 6.11 for sausage, Fig. S1).

Following the gastric phase, we noted significant differences in log reduction between sausage (mean log reduction 1.147) and cheese (mean log reduction 0.122, *p*-value < 0.0001), as well as between sausage and fish (mean log reduction 0.186, *p*-value < 0.0001, Table S1). During the gastric phase, we observed pH differences among the food matrices: for cheese, the pH shifted from 4.61 after the addition of gastric juice (pH 1.27) to 4.71 at the end of the gastric phase; for fish from 4.72 to 4.89; and for sausage from 3.96 to 4.07 (Fig. S1).

**Fig. 2:**
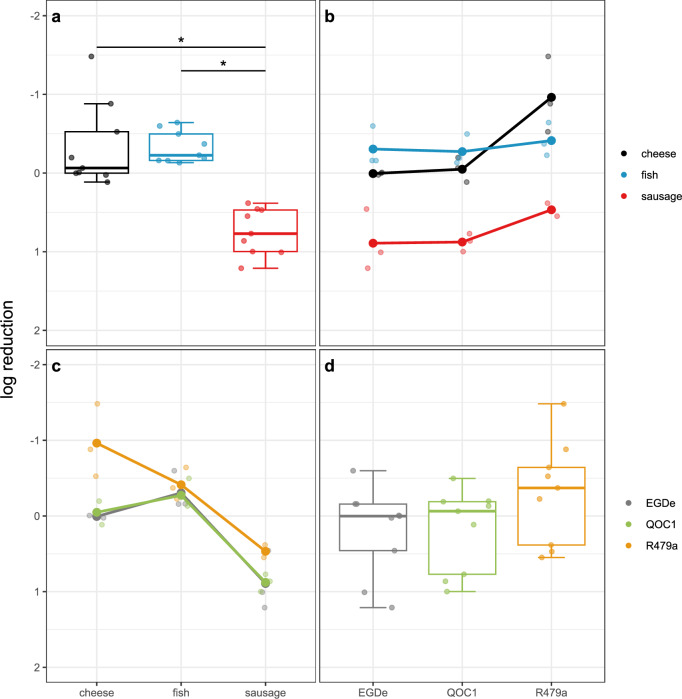
Survival of *L. monocytogenes* after the intestinal phase. Boxplot for log reduction split into the different food matrices (**a**) and the different strains (**d**). Interaction plot for log reduction showing interactions of the different food matrix depending on the strain (**b**) and of the different strains depending on the food matrix (**c**). Black: cheese (*n* = 9), blue: fish (*n* = 9), red: sausage (*n* = 9), grey: EGDe (*n* = 9), green: QOC1 (*n* = 9), orange: R479a (*n* = 9). *p < 0.05.

Upon comparing the log reduction after the intestinal phase, which represents the endpoint of our digestive model, we observed significant differences between sausage (with a mean log reduction of 0.7451) and both cheese (mean log reduction of -0.335, *p*-value = 0.0016) and fish (mean log reduction of -0.330, *p*-value = 0.0001, refer to Fig. 2). Interestingly, the bacteria preincubated in cheese and fish demonstrated a capacity for recovery, resulting in a higher count of culturable bacteria compared to the gastric phase. After the addition of duodenal juice (pH 7.87) and bile juice (pH 8.54), the pH levels were recorded as 6.71 for cheese, 6.79 for fish, and 7.00 for sausage. By the end of the intestinal phase, the pH levels were measured as 6.56 for cheese, 7.09 for fish, and 7.26 for sausage (Fig. S1).

### Invasion of *L. monocytogenes* surviving the intestinal phase

**Fig. 3:**
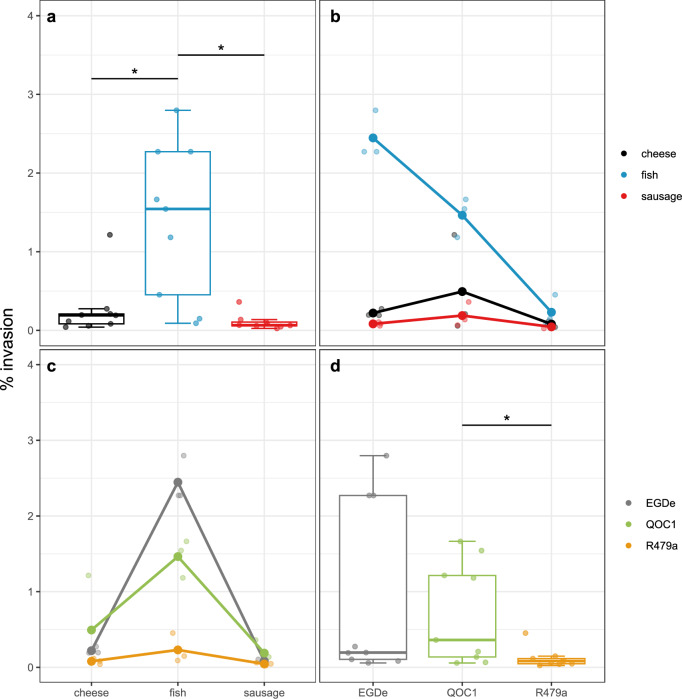
Invasion of *L. monocytogenes* surviving the intestinal phase. Boxplot for invasion efficiency (%) split into the different food matrices (**a**) and the different strains (**d**). Interaction plot for invasion efficiency (%) showing interactions of the different food matrix depending on the strain (**b**) and of the different strains depending on the food matrix (**c**). Black: cheese (*n* = 18), blue: fish (*n* = 18), red: sausage (*n* = 18), grey: EGDe (*n* = 18), green: QOC1 (*n* = 18), orange: R479a (*n* = 18). *p < 0.05.

In the final experimental step of our study, we infected human intestinal epithelial Caco-2 cells with *L. monocytogenes* that had been previously incubated in food and exposed to the GI tract model, and assessed the efficiency of bacterial invasion. Our analysis of the log-transformed percent invasion data revealed significant differences between bacterial strains (*p*-value = 0.0313) and food matrices (*p*-value = 0.0002, Fig. 3, Table S1). The invasion efficiency was defined as the percentage of bacterial cells, able to invade the host cell. Notably, *L. monocytogenes* preincubated in fish and exposed to the digestive model exhibited significantly higher invasion efficiency compared to bacteria preincubated in cheese (*p*-value = 0.0048) and sausage (*p*-value = 0.0001, Fig. 3a). Combining these data with the data from the survival of bacteria through the GI model, bacteria preincubated in fish resulted in the highest number of intracellular bacteria, followed by cheese. Preincubation in sausage resulted in the lowest number of intracellular bacteria. Further, the overall invasion efficiency of the strain R479a was significantly lower than that of QOC1 (*p*-value = 0.0486, Fig. 3d) across all food matrices, indicating a strain-dependent effect. No significant interactions between food matrices and strains were observed. The basal invasion efficiency (without food-GI stress conditions) of the three *L. monocytogenes* strains was additionally tested. The strain R479a displayed a significantly lower invasion efficiency than strain QOC1 (*p*-value = 0.0003, Fig. S4) and EGDe (*p*-value < 0.0001, Fig. S4).

### Gene transcriptional profiles of *L. monocytogenes*

**Fig. 4:**
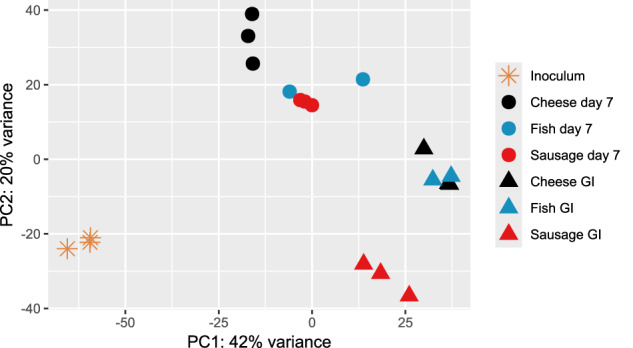
Principal component analysis (PCA) of *L. monocytogenes* transcriptomic data. Variance stabilizing transformation (vst) was performed before conducting PCA. Yellow star: Inoculum (*n* = 3); Black dot: after seven-day growth in cheese (*n* = 3); Blue dot: after seven-day growth in fish (*n* = 2); Red dot: after seven-day growth in sausage (*n* = 3); Black triangle: after passage through the GI model in cheese (*n* = 3); Blue triangle: after passage through the GI model in fish (*n* = 2); Red triangle: after passage through the GI model in sausage (*n* = 3).

RNA was extracted from EGDe of the inoculum, after its growth in the three food matrices for seven days at 10 °C and after exposure to the three-steps GI model. PCA indicated that growth in food matrices and GI stress were primary factors influencing changes in the *L. monocytogenes* transcriptome compared to the inoculum (Fig. 4). We identified two major clusters: one including the samples from the food matrices and another representing the post-GI stress conditions. Within these clusters, the type of food was an additional factor for transcriptome shifts.

### Gene transcriptional profiles in *L. monocytogenes* grown for seven days in sausage, fish and cheese

Numerous differences were noted in the gene expression profile of *L. monocytogenes* after seven-day growth across the three food matrices, especially when comparing the cheese samples to the fish and sausage samples. The number of significantly differentially expressed genes was 254 (up: 110/down: 144) between sausage and cheese, only 59 genes (up: 48/down: 11) between sausage and fish, and 286 (up: 136/down: 150) between fish and cheese (Tables S2–S4). Differentially expressed genes were assigned to 102, 88, and 43 KEGG pathways in fish versus cheese, sausage versus cheese, sausage versus fish, respectively (Figs.  S5–S7).

**Fig. 5:**
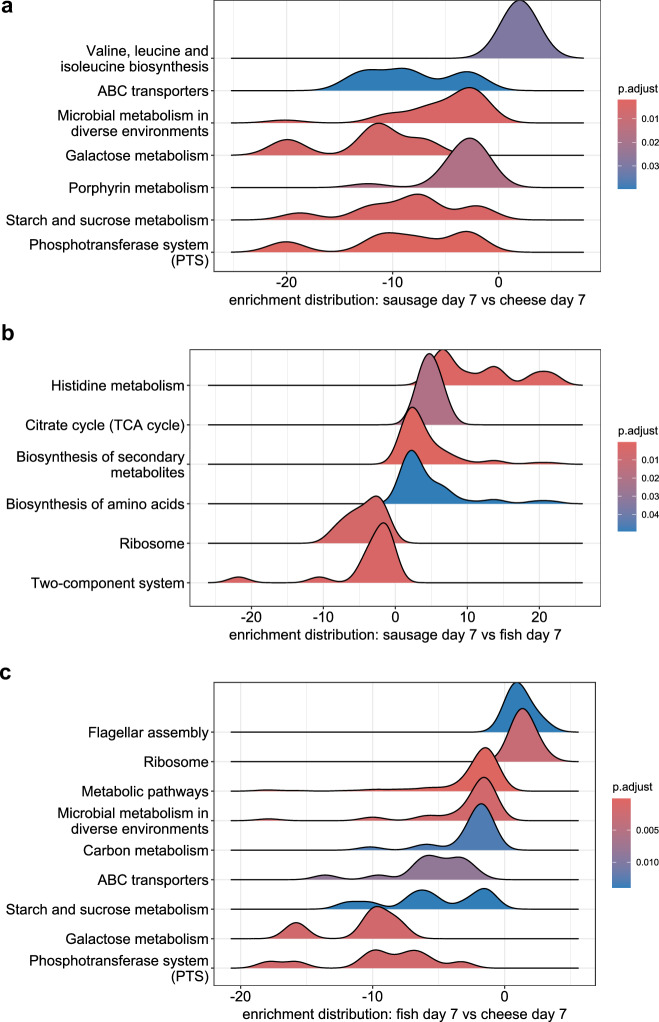
Gene set enrichment analysis (GSEA) for *L. monocytogenes* transcriptomic data after growth in food matrices for seven days. Ridgeplot showing enriched KEGG pathways (*p* adj < 0.05) in the full *L. monocytogenes* transcriptome after seven-day growth in sausage *versus* cheese (**a**), after seven-day growth in sausage *versus* fish (**b**), after seven-day growth in fish *versus* cheese (**c**).

With GSEA, enrichment of the upregulated pathway “Valine, leucine and isoleucine biosynthesis” and enrichment of the downregulated pathways “ABC transporters”, “Microbial metabolism in diverse environments”, “Galactose metabolism”, “Porphyrin metabolism”, Starch and sucrose metabolism”, and “Phosphotransferase system (PTS)” were identified when comparing sausage versus cheese (Fig. 5a, Table S5).

When comparing sausage versus fish, enrichment with positive log2FoldChange (log2FC) was observed for the pathways “Histidine metabolism”, “Citrate cycle (TCA cycle)”, “Biosynthesis of secondary metabolites”, “Biosynthesis of amino acids”, and enrichment with negative log2FoldChange was observed for the pathways “Ribosome” and “Two-component system” (Fig. 5b, Table S5). In the comparison between fish and cheese samples, the pathways ‘Flagellar Assembly’ and ‘Ribosome’ were significantly upregulated. On the other hand, the pathways ‘Metabolic Pathways’, ‘Microbial Metabolism in Diverse Environments’, ‘Carbon Metabolism’, ‘ABC Transporters’, ‘Starch and Sucrose Metabolism’, ‘Galactose Metabolism’, and ‘Phosphotransferase System (PTS)’ were significantly downregulated (Fig. 5c, Table S5).

### Effect of the GI stress on the gene transcriptional profile of *L. monocytogenes* grown in sausage, fish, and cheese

We subsequently analysed the effect of the GI model on the gene transcriptional profile of *L. monocytogenes* previously grown in the different food matrices (comparing the end of the intestinal phase to the 7-day 10 °C food matrix incubation timepoint). As *L. monocytogenes* in brain heart infusion broth did not survive the GI model, the effects of the GI conditions on the transcriptome of *L. monocytogenes* could not be investigated without the factor “food matrix”.

**Fig. 6:**
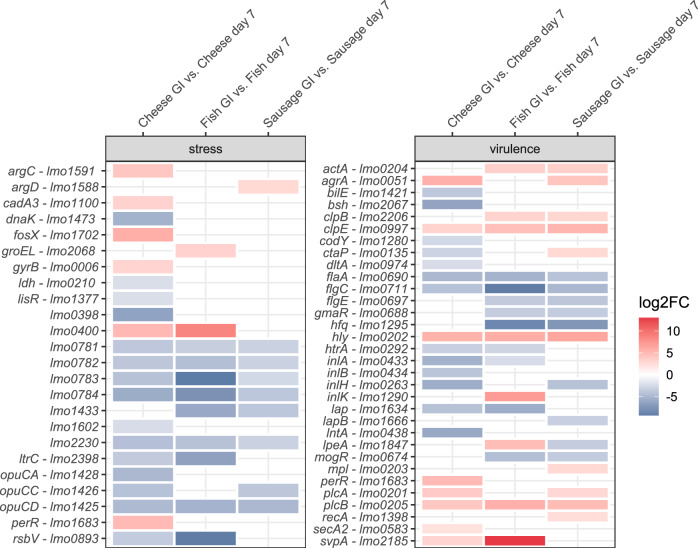
Differential gene expression of *L. monocytogenes* after GI stress in food matrices compared to growth in food for seven days. Changes in the gene expression of *L. monocytogenes* (*p* adj < 0.05, log2FoldChange ≥ |2|) of virulence and stress resistance genes in sausage, fish, and cheese after passage through the artificial GI model compared to growth in the three food matrices for seven days.

The passage through the GI model resulted in significantly differential gene expression of 238 genes (up: 100/down: 138) for sausage, 263 genes (up: 67/down: 196) for fish, and 509 (up: 158/down: 343) for cheese, respectively (Tables S6–S8). A detailed analysis of gene expression, including 95 stress- related and 99 virulence-associated genes (Table S9) revealed that 25 stress-related and 31 virulence genes were significantly differentially expressed (Fig. 6). Notably, *lmo2230*, involved in acid stress response^24^, and *opuCD*, associated with osmotic stress^25^, were downregulated in all three samples. In addition, the *lmo0781*-*lmo0784* operon, reported to be part of the σ^B^ regulon^26^, was significantly downregulated in all three food matrices at the end of the intestinal phase. *Lmo0400*, part of the general stress response^26^, was significantly upregulated in cheese and fish. In contrast, the cold stress gene *ltrC*^27^, and *rsbV*, component of the stressosome^28^, were downregulated. We have also identified five significantly upregulated stress genes in cheese: *argC, cadA3, fosX, gyrB, and perR*. Additionally, *groEL* was upregulated in fish, and *argD* in sausage.

In general, the gene transcription levels (TPM) varied in the different conditions (Table S10). E.g., *lmo2230*, *opuCD*, *lmo0781*, *lmo0782*, *lmo0783*, and *lmo0784*, were among the top 25% expressed genes in all three food matrices after growth for seven days, whereas *lmo0400* was only among the top 100-75% expressed genes in cheese and fish and among the top 75–50% expressed genes in sausage. *Lmo0781*, *lmo0782*, *gyrB* and *groEL* were among the top 25% expressed genes after GI conditions. In contrast, for *lmo0400* and *ltrC*, we observed low transcription levels (top 100-75%).

Three virulence genes were significantly upregulated in all three food matrices at the end of the GI model (Fig. 6). These genes include *clpE*, which is involved in intracellular survival^29^, and *hly* and *plcB*, both required for phagosomal escape of *L. monocytogenes*^30^. *ClpE*, *hly*, and *plcB* were among the top 25% or 50–25% expressed genes after GI conditions (Table S10). In contrast, the passage through the GI passage resulted in two significantly downregulated virulence genes in all food matrices, *flaA* and *flgC*, both involved in motility^31,32^. *FlaA* was highly transcribed (top 50–25%) and *flgC* was among the lower transcribed genes (top 75–50%, 100–75%) at the end of the GI model (Table S10). We also identified nine virulence genes that were significantly differentially regulated only in cheese (2 up/7 down) and three in sausage (2 up/1 down). Additionally, *inlK*, a medium to low transcribed gene in all conditions (top 75–50%, 100–75%), was found to be upregulated only in fish (Table S10).

A KEGG pathway analysis revealed genes assigned to 67 pathways as significantly differentially expressed in sausage after GI stress (Fig. S8), 105 pathways in fish and 124 in cheese (Fig. S9, Fig. S10). We identified by GSEA that the GI stress downregulated the pathways “Flagellar assembly”, “Pyrimidine metabolism” and “Biosynthesis of cofactors” in all three food matrices and the pathways “Alanine, aspartate and glutamate metabolism”, in sausage and cheese. In contrast, the upregulated pathways “Aminoacyl-tRNA biosynthesis” and “Valine, leucine and isoleucine biosynthesis” were enriched in cheese and fish due to GI stress. In cheese samples, we further identified the enrichment of the downregulated pathways “Phosphotransferase system (PTS)”, “Galactose metabolism”, “Starch and sucrose metabolism”, “Microbial metabolism in diverse environments”, and “Metabolic pathways”. In fish, exposure to the GI model additionally resulted in the enrichment of upregulated pathways “Biosynthesis of secondary metabolites”, “Carbon metabolism”, “Biosynthesis of amino acids”, “2-Oxocarboxylic acid metabolism”, “Pantothenate and CoA biosynthesis”, and “Histidine metabolism” and in the enrichment of the downregulated pathway “Two-component system” (Table S5).

### Effect of the food matrices on gene expression of *L. monocytogenes* after passage through the GI model

We also analysed the impact of the food matrices on *L. monocytogenes* gene expression after the passage through the GI model. In sausage, 127 genes were significantly differentially expressed (up: 115/ down: 12) compared to fish and 132 (up: 117/ down: 15) compared to cheese (Tables S11, S12). Interestingly, only 32 (up: 12/down: 20) significantly differentially expressed genes were identified between fish and cheese (Table S13).

**Fig. 7:**
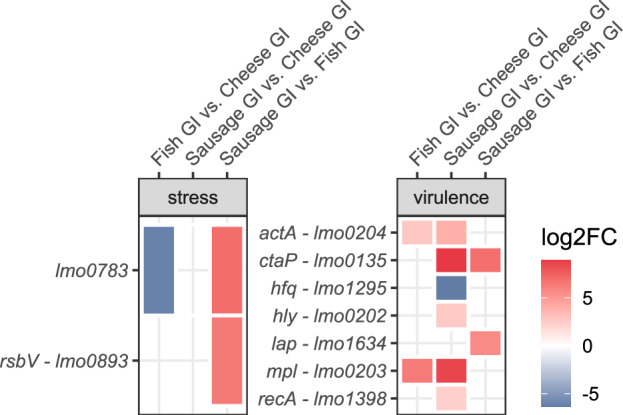
Effect of the food matrix on differential gene expression of stress and virulence genes of *L. monocytogenes* after GI stress. Differences in gene expression (*p* adj < 0.05, log2FoldChange ≥ |2|) of stress and virulence associated genes (custom-made stress and virulence related gene database) in *L. monocytogenes* in sausage, fish and cheese after the passage through the GI-model.

**Fig. 8:**
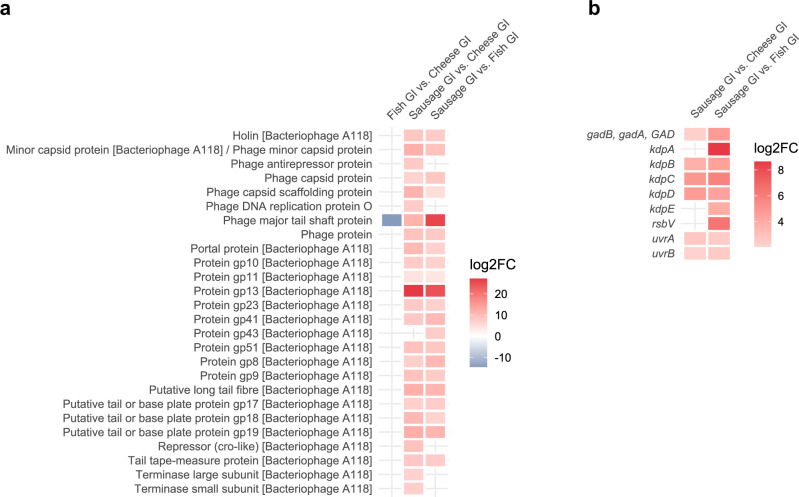
Effect of the food matrix on differential gene expression of *L. monocytogenes* after GI stress. Differences in gene expression (*p* adj < 0.05, log2FoldChange ≥ |2 | ) of bacteriophage genes (**a**) and of selected stress associated genes (KEGG annotation) (**b**) in *L. monocytogenes* in sausage, fish and cheese after the passage through the GI-model.

Gene expression analysis of 95 stress- and 99 virulence-associated genes (Table S9) revealed that only two stress-related and seven virulence genes were significantly differentially expressed between the food matrices (Fig. 7). *Lmo0783* required for oxidative stress response was significantly lower expressed in fish compared to the other two food matrices, in which it we observed high transcription levels (top 50–25%, Table S10). Conversely, *rsbV*, which is part of the stressosome^28^, was significantly higher expressed in sausage compared to fish. The overall transcription of *rsbV* was high in sausage (top 50–25%) (Table S10). The expression of the virulence genes *actA*, which is involved in cell-to-cell spread^30^, and *mpl* was significantly higher in fish and sausage compared to cheese after the GI stress. The transcription levels of *actA* were high in the food matrices (top 25%, 50-25%), whereas *mpl* was expressed at low levels in cheese and fish (top 100–75%, 75–50%) (Table S10). Moreover, we observed a higher expression of *ctaP* in sausage. *CtaP* is involved in host cell adherence^33^ and was among the top 25% expressed genes in sausage. In addition, we observed increased gene expression of 26 bacteriophage genes, DNA repair genes (*uvrA*, *uvrB)*^34^, the potassium homoeostasis genes *kdpA, kdpB, kdpC, kdpD, kdpE*, and a gene encoding for glutamate decarboxylase (GAD), involved in survival of gastric fluids^35^, after GI conditions in sausage compared to cheese and fish (Fig. 8). *UvrA*, *uvrB* and *kdpD* were among the top 25% expressed genes in all three conditions and most of the bacteriophage genes were among the top 25% or 50-25% expressed genes in sausage. In addition, flagellar-associated genes *fliS* and *flgB* were higher expressed in fish than in cheese or sausage.

Genes associated with 44 and 50 KEGG pathways were differentially expressed in sausage versus cheese and sausage versus fish after the passage through the GI model, respectively (Fig. S11, S12). When comparing fish to cheese samples, differential expression of genes assigned to 16 pathways was observed (Fig. S13). GSEA identified the “Ribosome” pathway as negatively enriched in sausage versus cheese after passage through the GI tract model. In sausage versus fish, the downregulated pathways “Histidine metabolism”, “Biosynthesis of amino acids”, and “Biosynthesis of secondary metabolites” were enriched. Furthermore, the “Biosynthesis of amino acids” pathway was additionally enriched in fish compared to cheese (Table S5).

## Discussion

Our study addressed the potential impact of different food matrices on the survival and virulence capacity of *L. monocytogenes* in the GI tract. This study used three RTE food matrices – “knackwurst” sausage, smoked salmon, and soft cheese, which are known to be frequently contaminated with *L. monocytogenes* and have been associated with human listeriosis cases^3^. Considering potential strain-dependent effects, we investigated the behaviour of three *L. monocytogenes* strains. The lab strain EGDe and two food isolates: Strain R479a (ST8), which was repeatedly isolated from smoked salmon^36,37^, and QOC1 (ST403), which was responsible for the “Quargel” cheese (soft cheese) listeriosis outbreak in 2009/2010^5,38^.

In the first step, we analysed the effect of three food matrices on the growth of three *L. monocytogenes* strains after incubation at 10 °C for seven days, which mimics the storage conditions in a household refrigerator. Our experiments mimic the situation that *L. monocytogenes* contamination occurs at the final production stage e.g., during slicing or packaging. However, *Listeria* contamination can occur at various stages in food production, from raw materials to finished products. Overall, the food matrices did not differentially affect growth, but we observed a strain-dependent effect. Strain-dependent effects on the growth of *L. monocytogenes* were observed, for example, in Greek traditional soft cheese at different temperatures during storage^39^, in milk and ham^40^, or in frankfurters^41^. The food matrices used in this study, all rich in lipids, may have promoted growth due to the presence of exogenous (unsaturated) fatty acids, which were reported to affect membrane fluidity and therefore support the growth of *L. monocytogenes* at cold temperatures^42,43^.

Our transcriptomic analysis showed that the food matrix altered the transcription of genes assigned to different metabolic pathways, reflecting the variability in available nutrient sources.

However, this study focuses only on one contamination time-point, namely, contamination at the final production stage. Contamination of the raw material or during earlier stages of production could impact the adaptive responses and behaviour of *L. monocytogenes*. For example, the co-occurring microbiota at the various production stages – in primary production or during food processing - can serve as a reservoir for exchanging genetic material such as conferring stress resistance genes^44–47^. Moreover, sublethal environmental conditions and stress during food processing can activate cross-protection responses such as temperature can impact the resistance to salt stress e.g., in the final product^48–50^.

Lactose served as the main carbon source for *L. monocytogenes* in cheese (0.5 g per 100 g). As a result, an increase in the transcription levels of genes associated with galactose metabolism, starch and sucrose metabolism, the phosphotransferase system (PTS), and ABC transporters, some of which are involved in sugar transport, was observed in cheese compared to sausage and fish. Positive enrichment of cobalt, nickel, and manganese transporters in cheese samples could indicate a higher demand for these metals in *L. monocytogenes* as they are important elements of many enzymes in bacteria^51,52^. A recent study observed higher gene expression of manganese transporters under sublethal acidic conditions. Furthermore, manganese uptake seems to promote the growth of *L. monocytogenes* in mild acid stress^53^. The mean value for pH in cheese was 5.91; but *L. monocytogenes* faces also in sausage and fish mild acidic conditions (pH 5.81 for sausage and 5.94 for fish). It remains unclear, if the increased expression of metal transporters is due to bacterial competition for these elements, as shown by another study^54^, or due to activation of metabolic pathways and enzyme activity requiring metals.

The growth in sausage led to the enrichment of upregulated gene sets that are involved in the biosynthesis of amino acids, particularly valine, leucine, and isoleucine. This may be necessary for maintaining membrane fluidity and promoting growth at low temperatures, as branched-chain amino acids have been found to influence the profile of fatty acids^55^. After seven-days growth in fish, transcription of genes associated with the ribosome was elevated, suggesting a higher general translation and protein synthesis rate. An increase in protein synthesis can be associated, for example, with the response to stress and adaptation, or even with the synthesis of virulence genes. A proteomic approach would be beneficial to comprehend the variations in the behaviour of *L. monocytogenes* in fish. Additionally, genes related to the two-component system showed increased expression in fish compared to sausage. The set of core enriched genes included different two-component systems, such as the operon *dltABCD* conferring resistance against food antimicrobials^56^, the *agr* operon components *agrA* and *agrB*, which are involved in virulence^57^, multiple transcriptional regulation mechanisms^58^ and biofilm development^59^, and *cheA*/*cheY*, which were reported to support the invasion of *L. monocytogenes*^31^. Further, genes associated with flagellar assembly were enriched for higher transcript levels after growth in fish, contributing to virulence^31^. The reason for higher expression of flagella genes might be the exposure to sodium lactate, used as an acidity regulator in smoked salmon, as reported in previous research^60^.

The passage through the human GI tract represents a critical step in the infectious process, where *L. monocytogenes* encounters an acidic environment in the stomach and duodenum, and the presence of bile in the duodenum^9^. Overall, we observed that most bacteria survived the exposure to the GI stress (max. mean log reduction 1.147 in sausage after the gastric phase).

The food matrices were a mild acidic growth environment for *L. monocytogenes*, with a mean pH value of 5.81 for sausage, 5.91 for cheese and 5.94 for fish, respectively. Therefore, the adaptive acid tolerance response has been induced in our experiments, which protected *L. monocytogenes* from the pH stress during the gastric stress^61,62^. This is in line with our observation that *L. monocytogenes* in broth did not survive the artificial GI model. To assess the influence of GI conditions on the transcriptome of *L. monocytogenes* in the three food matrices, we compared the effect of GI stress on gene expression in strain EGDe (after the GI model versus seven-day 10 °C growth) focusing on stress resistance and virulence genes.

*ArgC*, which is involved in acid resistance^63^, was higher expressed in the cheese samples, which reflects the slightly acidic conditions in cheese (pH 6.56) at the end of the intestine phase (in contrast, the pH value in the fish samples was 7.09, and in sausage 7.26). Expression of *argD*, additionally involved in acid resistance^63^, was increased after GI stress in sausage, probably due to low pH in sausage during the gastric phase. Downregulation of *ltrC*, which promotes growth at low temperatures^27^, correlated with the higher temperature during the GI model (37 °C) compared to storage at 10 °C. Osmotic stress was likely higher during the growth in food matrices, especially in cheese samples, than at the end of the GI model, since several genes related to osmotic stress were downregulated (*opuCA*, *opuCC*, *opuCD*). The salt content in the food was higher than in the GI model. Additionally, the decrease in expression of the *opuC* operon may also be affected by the temperature change from 10 °C during storage to 37 °C during the GI model, as *opuC* has also a role in cold stress adaptation^64^. Differences in the gene regulation of stress resistance genes between the three food matrices after GI stress could also be caused by the variety of carbon sources e.g., by lactose in cheese. A study reported that lactose availability induces *σ*^*B*^-dependent stress response and increases resistance to acid stress and heat stress^65^. The expression of flagella genes was decreased after exposure to GI stress at 37 °C in all three matrices, consistent with literature as flagella genes are only expressed at lower temperatures^32^. Surprisingly, *inlA* expression was downregulated after GI stress in fish and cheese. Analysis of the protein levels of InlA would be required to gain further insights as we only analysed gene expression at the end of the GI model. The expression of the virulence-associated *svpA* gene was increased in cheese and fish samples. Lower iron availability in cheese and fish after GI stress, but not in sausage, may have induced higher transcription of *svpA*^66^. GI stress in fish increased the expression of the virulence factors *inlK* and *lpeA*, explaining the higher invasion efficiency of *L. monocytogenes* in the fish samples, as *inlK* promotes virulence by escaping autophagy during infection^67^, and *lpeA* boosts the entry into host cells^68^. As *Listeria* did not survive the GI model without the protective effects of the food matrix, the effects of the GI conditions on the transcriptome of *L. monocytogenes* could not be investigated without the factor “food matrix”. It remains for future studies to investigate how transcriptomic changes are impacted by GI conditions or food stress alone.

We observed that *L. monocytogenes* had a higher survival rate in cheese and fish after the gastric phase compared to sausage. As a result, the number of bacteria was also higher in fish and cheese after the intestinal phase compared to sausage. Additionally, *L. monocytogenes* was able to recover in both fish and cheese during the intestinal phase. Our results indicated that the different pH during the gastric phase in the food matrices caused differences in survival. Sausage showed the lowest buffering capacity as the pH ranged from 3.96 after the addition of gastric juice (pH 1.27) to pH 4.07 at the end of the gastric phase in sausage compared to 4.61 to 4.71 in cheese and 4.72 to 4.89 in fish. At the end of the intestinal phase, the pH was 6.56 for cheese, 7.09 for fish, and 7.26 for sausage. The significantly lower buffer capacities in sausage could be due to the fat and protein content. A recent study demonstrated that proteins in food protect *L. monocytogenes* from GI stress^69^. The protein content in sausage (11 g/100 g) was lower compared to cheese (17 g/100 g) and fish (21 g/100 g) in our study. Moreover, fat seems to have a protecting effect. Barmpalia-Davis et al. reported that high fat content in beef frankfurter protected *L. monocytogenes* from acidic conditions during a simulated dynamic digestion^70^. However, the fat content of 24 g/100 g in sausage (compared to 31 g/100 g in cheese and 10 g/ 100 g in smoked salmon) could not compensate for the low protein content.

Comparison of gene expression profiles after passage through the GI model between fish, cheese and sausage revealed that several stress-related genes were upregulated in sausage, which is in line with the survival data. In response to the poor buffer capacities of sausage and the pH alterations, genes involved in resistance to acid stress (*gadA*, *gadB*)^35^ were upregulated. Further, the hostile conditions induced gene expression of the general stress response (*rsbV*)^28^, as well as several bacteriophage genes of the prophage A118, which is located within the *comK* gene. Expression of holin and endolysin suggested that host cell lysis occurred, resulting in bacterial cell death^71^. Therefore, bacteriophage activity could be an additional factor leading to fewer living bacteria in sausage. Increased transcription of prophage genes via SOS response was already observed e.g., when *L. monocytogenes* were treated with acids^72,73^. We further observed increased expression of DNA repair genes in sausage, which is in line with previous research, showing higher expression of the DNA repair genes *recA*, *uvrA* and *uvrB* in acid and bile stress conditions^34,74^. Upregulation of genes involved in potassium homoeostasis (*kdpA*, *kdpB*, *kdpC*, *kdpD*, *kdpE*) and *lmo0783* occurred potentially due to higher osmotic stress conditions in sausage. The increased transcription of stress resistance genes showed that *L. monocytogenes* in sausage faced more stress, leading to impaired bacterial replication and growth, thereby contributing to the decreased recovery of viable culturable bacteria counts after the intestinal phase^75,76^. This study used the static in vitro digestion model of Versantvoort et al.^77^. However, a variety of digestion models exists, making comparison between studies challenging^78^. The current state-of-the-art protocol for static in vitro digestion is the standardized INFOGEST 2.0 protocol based on an international consensus^79^, which was unfortunately published after the start of our project. This protocol has already been used in *Listeria* research^80–85^; however so far only in six studies (of which only two used *Listeria* contaminated food). In future studies we will use the simulator of the human intestinal microbial ecosystem (SHIME^®^), which is a dynamic model of digestion in accordance with the INFOGEST 2.0 protocol with the ability to mimic the human intestinal ecosystem with high dynamics and stability^86^.

In the last step of the food-GI tract-infection model, we examined the invasion efficiency of *L. monocytogenes* that survived the GI model. Unexpectedly, we observed a significantly higher invasion efficiency of *L. monocytogenes* after preincubation in fish and exposure to the gastrointestinal model for all three strains combined. Furthermore, we observed strain-dependent differences in our model. Similar differences among the single strains on the invasion were observed when we investigated the basal invasion efficiency without applying stress conditions on *L. monocytogenes*. Therefore, we concluded the invasion efficiency is influenced by both the type of strain and by the food matrix. Food-specific factors such as pH, salt concentration, or organic acid concentration are suggested to influence the invasion potential of *L. monocytogenes.* For example, Garner et al. reported higher invasion efficiency in the presence of salt or sodium lactate^87^. Given that the smoked salmon used in our study contained salt and sodium lactate as an acidity regulator, this could potentially explain the increased invasion of *L. monocytogenes* preincubated in fish. However, we did not measure the salt and sodium lactate content at the end of the GI phase.

In addition to sodium lactate, the fat content and composition could be significant factors. Among all three food matrices, fish has the lowest fat content (10 g/100 g compared to 24 g in sausage and 31 g in cheese), and the fatty acid composition varied greatly across the food matrices. However, data on the effect of fat on pathogenicity are limited. A recent study by Las Heras et al. reported that a short-term high-fat diet significantly increased susceptibility to *L. monocytogenes* infection in a mouse model, due to an increase in goblet cells and changes in the intestinal microbiota^88^. In contrast, Mytle et al., observed that the relative fat content of dairy products did not affect the *L. monocytogenes* numbers in a mouse infection model, suggesting that the type of fat, specifically the fatty acid composition, plays a crucial role^89^. Certain fatty acids are known to influence the invasion efficiency of *L. monocytogenes* in Caco-2 cells^90^. Long-chain free fatty acids, rarely present in smoked salmon, can act as signals to prevent PrfA-mediated activation of virulence genes^91^. C_18_ unsaturated fatty acids were further reported to inhibit PrfA, resulting in decreased transcription of virulence factors. The effect was observed for e.g., linolenic acid, linoleic acid and oleic acid^92,93^, as well as for C_16_ palmitoleic acid^91^, which were either not detected or only found in low amounts in fish in this study (linolenic acid: 0 µg/ml, linoleic acid: 9.73 µg/ml, oleic acid: 27.58 µg/ml, palmitoleic acid: 2.24 µg/ml). Overall, the content of these fatty acids was higher in sausage (linolenic acid: 0 µg/ml, linoleic acid: 21.79 µg/ml, oleic acid: 104.58 µg/ml, palmitoleic acid: 6.53 µg/ml) and cheese (linolenic acid: 1.5 µg/ml, linoleic acid: 5.59 µg/ml, oleic acid: 75.92 µg/ml, palmitoleic acid: 6.36 µg/ml) and might cause the reduced virulence of *L. monocytogenes*. The upregulation of two flagella genes (*fliS*, *flgB*) in fish may have supported invasion into Caco-2 cells, as flagella-mediated motility has been reported as an important factor for invasion into epithelial cells^94^. In addition, an unknown gene (coding sequence 3_123), which is part of the flagella operon, was significantly higher expressed in fish compared to sausage and cheese. The gene codes for a hypothetical transmembrane protein containing a (predicted) signal peptide sequence. Further characterization of this gene would be required to obtain knowledge on its function in virulence. Notably, genes involved in the biosynthesis of amino acids were enriched in the transcriptome of EGDe in fish after GI stress. The availability of amino acids is essential for protein translation, bacterial growth and virulence.

Modulating the fat and protein content in food could be one potential way to decrease the survival abilities of *L. monocytogenes* in high-risk food in order to improve food safety. The results of our study further suggest that the properties of smoked salmon in combination with the GI conditions increased virulence. Future research could reveal the mechanism behind this effect, which can be used for improving food safety applications.

In conclusion, the type of food plays a significant role in survival, virulence, and transcriptome of *L. monocytogenes* within a food-gastrointestinal-infection model. Our results demonstrates that the food matrix has a direct impact on the behaviour and pathogenic potential of *L. monocytogenes*, highlighting the importance of understanding these interactions for food safety and public health.

## Methods

### Bacterial strains

Experiments were performed using the *L. monocytogenes* lab strain EGDe (serovar 1/2a, sequence type (ST)35), the “Quargel”-cheese outbreak strain QOC1 (Austria, serovar 1/2a, ST403^38,95^, and the fish (smoked salmon) associated isolate R479a (Denmark, serovar 1/2a, ST8)^36,37^. The whole experimental set-up is graphically shown in Fig. S14.

### Preparation and inoculation of food

“Brie” soft cheese, smoked salmon and heat-treated non-fermented sausage (“knackwurst”) were purchased from a local supermarket. Ingredients and nutritional information were obtained from the product packaging (Table S14). Different batches of food were used. *L. monocytogenes* was not detected in the purchased food matrices. For determining the fatty acid profiles of the three food matrices, the samples were freeze-dried and transesterified using trimethylsulfonium hydroxide according to DGF standard method (C-VI 11e (18)). Fatty acids were analysed with gas chromatography coupled with a flame ionization detector (Fisons 8000 Series) on a Restek RTX-225: 30 m, 0.25 mm ID, 0.25 µm df column. As an internal standard, the Supelco 37 comp FAME-Mix was used.

For the food-GI-infection model, the cheese rind was removed, and the knackwurst sausage was peeled. Cheese, sausage and smoked salmon were cut in small pieces of approximately 2 ×1.5 cm size and minced in a food chopper (Ultimate Chopper) for 10 s. Minced food was stirred by using a spatula and was chopped again for 10 s. Then, 50 g aliquots of each food matrix stored in Stomacher filter bags (Seward) were frozen at −20 °C until further use.

A single colony of *L. monocytogenes* strains grown on tryptic soy agar complemented with yeast extract (TSA-Y, BIOKAR diagnostics) was inoculated in 8 ml brain- heart infusion with yeast extract (BHI-Y, BIOKAR diagnostics) and cultivated for 7 h at 37 °C shaking (125 rpm). The optical density (OD_600_) of bacterial cultures was then adjusted to 0.1 in 8 ml BHI-Y and the bacteria were cultivated at 10 °C for approximately 40 h (pre-adaption).

The food matrices (50 g) were thawed at room temperature, inoculated with 10^6^ colony forming units per gram (CFU g^-1^) food (inoculum volume of 40–70 µl), blended using the laboratory blender Stomacher 400 (Seward) at middle speed for 60 s and incubated for 7 days at 10 °C. Each food matrix was inoculated separately with each *L. monocytogenes* strain used in this study. The inoculum level was determined by plating serial dilutions on TSA-Y plates and incubating them for 48 h at 37 °C.

### Determination of bacterial growth in food

To investigate bacterial growth in “Brie” cheese, smoked salmon and “knackwurst” sausage after 7 days at 10 °C, 200 ml Buffered Peptone Water (Oxoid) was added. Samples were blended using the laboratory blender Stomacher 400 (Seward) at normal speed for 60 s and serial dilutions were plated on PALCAM (BIOKAR diagnostics) agar plates in triplicates to determine CFU per g food (taking the total volume of food matrices and peptone water into consideration). The log increase was calculated by applying the following Eq. (1):

1 $$Log\,increase=\log\left({\mathrm{CFUg}}_{7{\mathrm{days}},{10}^{\circ}{\rm{C}}}^{-1}\right)-{\log}\left(CFU{g}_{inoculum}^{-1}\right)$$

### Determination of *L. monocytogenes* survival after oral, gastric and intestinal phases

The digestive juices (artificial saliva, gastric juice, duodenal juice and bile juice) were prepared according to Versantvoort et al. (Table S15) and stored at −20 °C until further use. As this study started before the publication of the INFOGEST 2.0 protocol, we followed the protocol of Versantvoort et al.^77^, which was among the most highly cited approaches. The digestive juices were thawed at 37 °C in a water bath. In the oral phase, 33.33 ml artificial saliva was added to each infected food matrix and incubated for 7 days at 10 °C. The food-saliva mixture was homogenised using the laboratory blender Stomacher 400 (Seward) at middle speed for 60 s. Subsequently, in the gastric phase, 66.66 ml gastric juice was added to the oral bolus. Again, the food-digestive-fluid mixture was homogenised using the laboratory blender Stomacher 400 (Seward) at normal speed for 60 s and incubated for 2 h at 37 °C shaking (125 rpm). In the intestinal phase, 66.66 ml duodenal juice, 33.33 ml bile juice and 11.11 ml NaHCO_3_ (stock concentration 84.7 g/l) were added, and the samples were incubated for a further 2 h at 37 °C shaking (125 rpm). After the oral, gastric, and intestinal phase, an aliquot of 50 µl was taken for CFU determination. Therefore, serial dilutions were plated on PALCAM (BIOKAR diagnostics) agar plates in triplicates and incubated for 48 h at 37 °C. The log reduction after oral, gastric and intestinal phases was calculated using the following Eq. (2):

2 $$Log\,reduction={\log}\left({\mathrm{CFUg}}_{7{\mathrm{days}},{10}^{\circ}{\rm{C}}}^{-1}\right)-{\log}\left({\mathrm{CFUg}}_{\mathrm{oral}/\mathrm{gastric}/\mathrm{intestinal}\,\mathrm{phase}}^{-1}\right)$$

The different volumes were taken into consideration for the calculation of the CFU g^-1^ food.

The pH values of the food, the digestive juices and during the digestive phases were determined using the Fisher Scientific Accumet AE150 pH metre.

### Determination of the invasion efficiency of *L. monocytogenes* surviving the intestinal phase

Human intestinal epithelial Caco-2 (ATCC® HTB­37™) cells were cultured in Eagle’s minimum essential medium (MEM/EBSS; Fisher Scientific) containing 10% foetal bovine serum (FBS; HyClone), 1% penicillin/streptomycin (HyClone) and 1% non-essential amino acids (Fisher Scientific) in non-coated cell culture flasks at 37 °C in 95% humidity and 5% CO_2_. For in vitro invasion assays, Caco-2 cells were seeded in 24-well plates in MEM/EBSS including 10% FBS, 1% penicillin/streptomycin and 1% non-essential amino acids prior to experiments to achieve a minimum confluence of 80%. Medium in 24-well plates was changed to MEM/EBSS containing 10% FBS without antibiotics 4 h before infection. In addition, the cell count per well in 24-well plates was determined.

After the intestinal phase, samples were transferred into 50 ml centrifugation tubes and centrifuged at 1555 × *g* at 37 °C for 5 min. The middle supernatants were transferred to new 50 ml tubes and centrifuged at 3220 × *g* at 37 °C for 5 min. The pellet was washed with 10 ml PBS, centrifuged at 1555 × g at 37 °C for 5 min, and resuspended in 10 ml pre-warmed (37 °C) MEM/EBSS including 10% FBS without antibiotics. Caco-2 monolayers were infected with *Listeria* at a multiplicity of infection of 25 for 1 h at 37 °C in 95% humidity and 5% CO_2_. Three wells were infected per condition. The number of bacteria used for Caco-2 infection was determined by plating serial dilutions on PALCAM (BIOKAR diagnostics) agar plates in triplicate. After the infection, Caco-2 cells were washed twice with 1 × PBS to remove non-adhering *L. monocytogenes* and subsequently, incubated for 45 min in MEM/EBSS containing 10% FBS and 100 µg/ml gentamicin (Sigma-Aldrich) at 37 °C. Cells were washed twice with 1 × PBS and lysed using cold 0.1% Triton X-100 (Merck). CFUs were determined by plating serial dilutions on PALCAM agar plates in triplicates which incubated for 48 h at 37 °C. Invasion (%) was calculated using the following Eq. (3):

3 $$\frac{{{\rm{CFU}}}_{45\min {\rm{gentamicin}}}}{{{\rm{CFU}}\left({\rm{mean}}\right)}_{{\rm{inoculum}}}}$$

### Assessment of the basal invasion ability of *L. monocytogenes*

Single colonies of *L. monocytogenes* EGDe, QOC1 and R479a were inoculated in in 8 ml brain-heart infusion with yeast extract (BHI-Y, BIOKAR diagnostics) and cultivated over-night at 37 °C shaking (125 rpm). Caco-2 cells were cultivated in 24-well plates in MEM/EBSS (Cytiva HyClone) including 10% FBS, 1% penicillin/streptomycin and 1% non-essential amino acids prior to experiments to achieve a minimum confluence of 80%. Medium in 24-well plates was changed to MEM/EBSS containing 10% FBS without antibiotics 4 h before infection. Cell count per well in 24-well plates was determined before conducting the invasion assay. Two hours prior to the experiment, over-night *L. monocytogenes* cultures were adjusted to OD_600_ 0.1, and bacteria were incubated at 37 °C shaking (125 rpm). For the invasion assay, Caco-2 cells were infected with a multiplicity of infection of 25 for 1 h. Subsequently, cells were washed twice with 1 × PBS followed by incubation in MEM/EBSS containing 10% FBS and 100 µg/ml gentamicin (Sigma-Aldrich) at 37 °C for 45 min. Cells were washed twice with 1 × PBS and cell lysis was conducted with cold 0.1% Triton X-100 (Merck). Serial dilutions of the bacterial inocula and the bacteria that successfully invaded Caco-2 cells were plated out on tryptic soy agar complemented with yeast extract (TSA-Y, BIOKAR diagnostics). Invasion (%) was calculated using the following Eq. (4):

4 $$\frac{{{\rm{CFU}}}_{45\min {\rm{gentamicin}}}}{{{\rm{CFU}}\left({\rm{mean}}\right)}_{{\rm{inoculum}}}}$$

### Statistical analysis of survival data

Each experiment was performed independently three times (three biological replicates) including three technical replicates for the growth (log increase) and survival (log reduction) and six technical replicates for the invasion efficiency.

For each dataset (i.e., log increase, log reduction after the oral, gastric and intestinal phase, and invasion efficiency), a one-way ANOVA was applied to evaluate the importance of the variability of the technical replicates. As the variability of the technical replicates was not significant, the means of the technical replicates were used for further analysis. The datasets were checked for normality with histograms, Q-Q plots and Shapiro-Wilk-test. The homoscedasticity was analysed with the Levene test. To perform inference statistics, the percentage invasion data was normalized using a log transformation. Normally distributed data with homogeneous variances was investigated with a one-way ANOVA followed by a Tukey Honest Significant Differences test. Not normally distributed data were analysed with the Kruskal-Wallis rank sum test, followed by a Dunn test (package “dunn_test” v1.3.5) with Bonferroni alpha adjustment. Details (n values, mean, standard deviation, statistical tests, p-values) on the statistical tests for each data set are summarised in Table S1. All analyses were conducted using the open-source statistical computing environment R v.3.5.3^96^.

### Total RNA extraction and RNA sequencing

For RNA extraction, 200 ml of Buffered Peptone Broth (Oxoid) was added to each food sample inoculated with *L. monocytogenes* strain EGDe and incubated for 7 days at 10 °C. Subsequently, the samples were blended using a laboratory blender (Stomacher 400, Seward) at medium speed for 60 s. The resulting food-liquid mixture was transferred to four 50 ml Falcon conical tubes per sample, which were then centrifuged at 3220 × *g* at 10 °C for 5 min. The supernatant was transferred to new 50 ml tubes. At this point, only the liquid part of the supernatant was transferred, avoiding the transfer of any food matrix particles. The samples were centrifuged at 4600 x g at 10 °C for 15 min and the supernatants were discarded. Additionally, for isolation of *L. monocytogenes* RNA in “Brie” soft-cheese, 40 ml of a lysis buffer, including 2 M MgCl_2_, 50 mM Tricine and 1% Lutensol^97^ in distilled water, were added to each pellet on ice. Samples were shaken rigorously for 1 min by hand followed by centrifugation at 4600 × *g* at 4 °C for 15 min.

In addition, the (bacterial) pellets harvested from the inoculum and after the intestinal phase, were subjected to RNA isolation.

Total RNA isolation was conducted using the RNeasy PowerMicrobiome Kit (Qiagen). The pellets were suspended in 650 µl solution PM1 and 6.5 µl beta-mercaptoethanol. Pellets of each food matrix sample, respectively, were pooled during the suspension step. Then, samples were transferred to PowerBead tubes (glass 0.1 mm) as described in the manufacturer’s instructions. Bead-beating was done at a speed of 4.5 m/s for 45 s (FastPrep Bead Beater, MP Biomedicals). Subsequently, the samples were cooled on ice for 2 min. The bead beating procedure was repeated five times. The subsequent RNA isolation steps were performed according to the manufacturer’s instructions with minor modifications. The final elution step was performed with 50 µl RNAse-free water and 1 µl Ribolock was added immediately. The remaining DNA was removed from the extracted RNA with the Turbo DNA-Free kit (Invitrogen). 50 µl RNA were mixed with 3 µL DNase (Invitrogen) and 3 µl DNase buffer and incubated for 30 min at 37 °C. Then, 11 µl inactivation reagent (Invitrogen) was added and the samples were centrifuged for 5 min at 10,000 × *g*. The supernatant was transferred to a new tube and samples were stored at −80 °C.

### RNA sequencing and bioinformatics

RNA of three biological replicates was shipped on dry ice to Vienna BioCenter Core Facilities GmbH for sequencing. RNA samples underwent standard Illumina library preparation utilizing the NEBNext® UltraTM RNA Library Prep Kit from Illumina. The removal of rRNA was performed using the Ribo-ZeroTM Magnetic Gold (Epidemiology) Kit developed by Epicentre Biotechnologies. Double-stranded cDNA libraries were created and paired-end reads were generated using an Illumina NovaSeq system. The quality of the raw sequencing data (average length = 50 bp) was first evaluated using FastQC v0.11.9^98^ and MultiQC v1.11^99^. Residual adapters were removed and quality reads with a Phred score below 25 and a minimum length of 18 bp were removed by using Trimmomatic v0.39^100^. Reads aligning to PhiX or the human genome (hg19) were discarded by using Bowtie2 v2.4.2^101^ and potential rRNA reads were discarded using SortMeRNA v4.3.3^102^. The quality-controlled sequencing reads resulted in an average of 9 million paired-ends per sample and were then aligned against the reference EGDe genome by using Bowtie2. To get the genomic features from the EGDe reference genome, it was submitted to the TORMES v1.3.0 pipeline^103^, by enabling Prodigal v2.6.3^104^ and using the KEGG database^105^. The resulting GFF annotation file was used to correct the resulting read counts of the RNAseq data against the EGDe genome by using featureCounts v2.0.1^106^. The resulting gene counts were normalized to Transcript Per Million (TPM) (Table S5) using the formula described by Zhao et al^107^. In addition, a custom-made database including genes associated with stress resistance and virulence in *L. monocytogene*s (Table S14) was created and screened by using DIAMOND v2.0.15^108^. Predicted coding sequences of the reference genome were then annotated using blastp implemented in DIAMOND using the fast sensitivity mode with a minimum sequence identity of 80%. Only sequences with an alignment length of over 90% were considered for further analysis.

Initial exploration of the transcriptomic dataset was conducted with R packages dplyr v1.1.4^109^ and tidyr v1.3.1^110^. Samples with low mapping rates against the reference genome were excluded from further analysis. Therefore, three biological replicates of *L. monocytogenes* after growth for seven days and survival of the GI-model in sausage and cheese, and two biological replicates of *L. monocytogenes* after growth for seven days and survival of the GI-model in fish were included in further analyses. Differential gene expression was explored using DESeq2 v1.42.1^111^. For conducting a principal component analysis (PCA), a variance stabilizing transformation was done using the “vst” function in DESeq2. Coding sequences with a Benjamini-Hochberg adjusted (“BH”) *p*-value < 0.05 and a log2FoldChange of > |2| were considered as significantly differentially expressed. Annotation of KEGG pathways was performed using the “bitr_kegg” function in the ClusterProfiler package v4.11.0^112^. Annotation of “lmo” gene identifiers to K-numbers (KEGG database) was done using a custom-made bash script accessing the KEGG API. For gene set enrichment analysis (GSEA), the “lfcShrink” function in the DESeq2 package^111^ in combination with the apeglm package v1.24.0^113^ was applied to obtain shrunken log2FoldChange values as input data. The total transcriptome was used as input data for GSEA, i.e., no *p*-value or log2foldChange threshold was applied. Genes were ranked by multiplying the -log10 transformed *p*-values with the algebraic sign of the log2FoldChange. GSEA and visualization were performed with ClusterProfiler v4.11.0^112^ and DOSE v3.28.2^114^. A seed was set (1234) and a cut-off value of 0.05 for the *p*-value with Benjamini-Hochberg (“BH”) *p*-value adjustment was applied for GSEA. Additional figures were created with ggplot2 v3.5.0^115^.

## Supplementary information

S1-S14_Fig

S1-S15_Tables

## Data Availability

The RNA sequencing raw data generated and analysed during the current study are available in the NCBI repository (BioProject number PRJNA1153315).
