# Supplementary material for: The type of food influences the behaviour of Listeria monocytogenes in a food-gastrointestinal-infection model
Source: NPJ Sci Food. 2025 May 19;9:79. doi: 10.1038/s41538-025-00436-5 (PMC12089613; doi:10.1038/s41538-025-00436-5)
Supplement: Supplementary file 1 — S1-S14_Fig [file 41538_2025_436_MOESM1_ESM.pdf]

Supplementary Material

Supplementary Figures

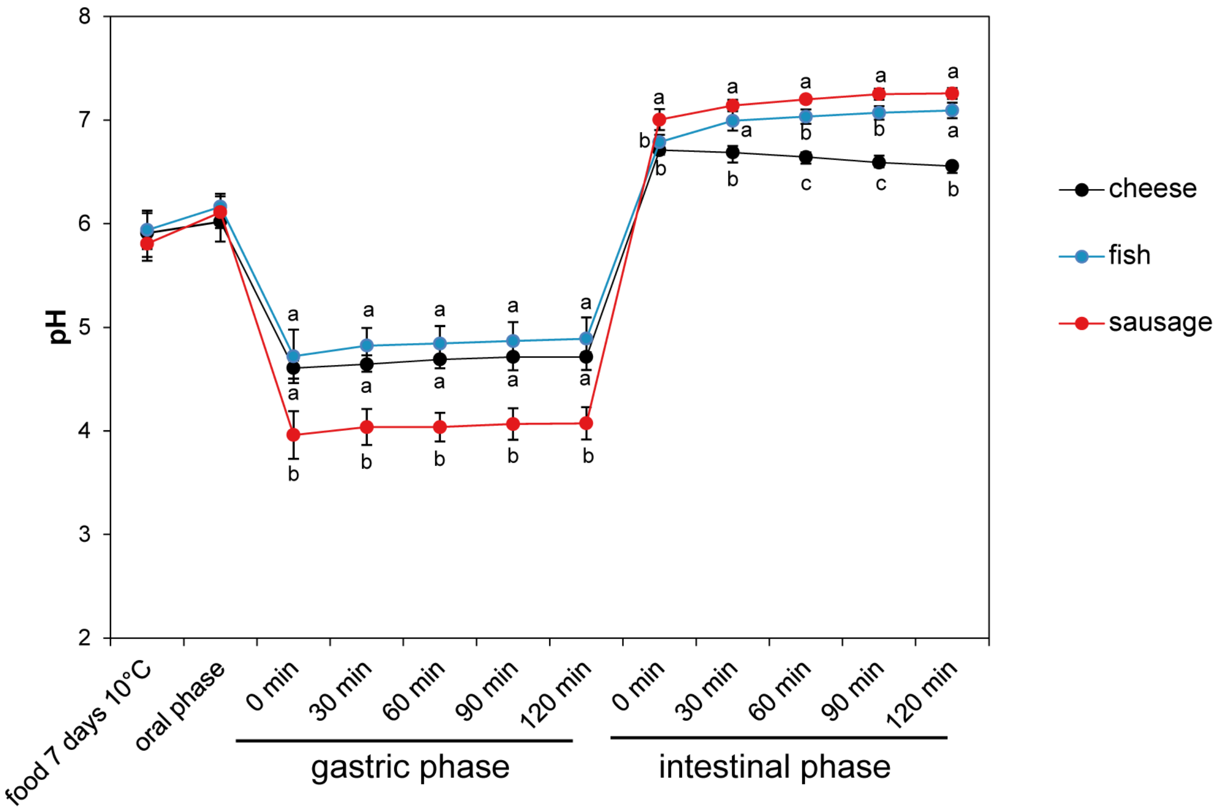

S1 Figure. pH values at different timepoints of the food-GI-infection model. Different letters (a, b, c) indicate statistically significant differences ($p < 0.05$) among the food matrices (cheese, fish, sausage).

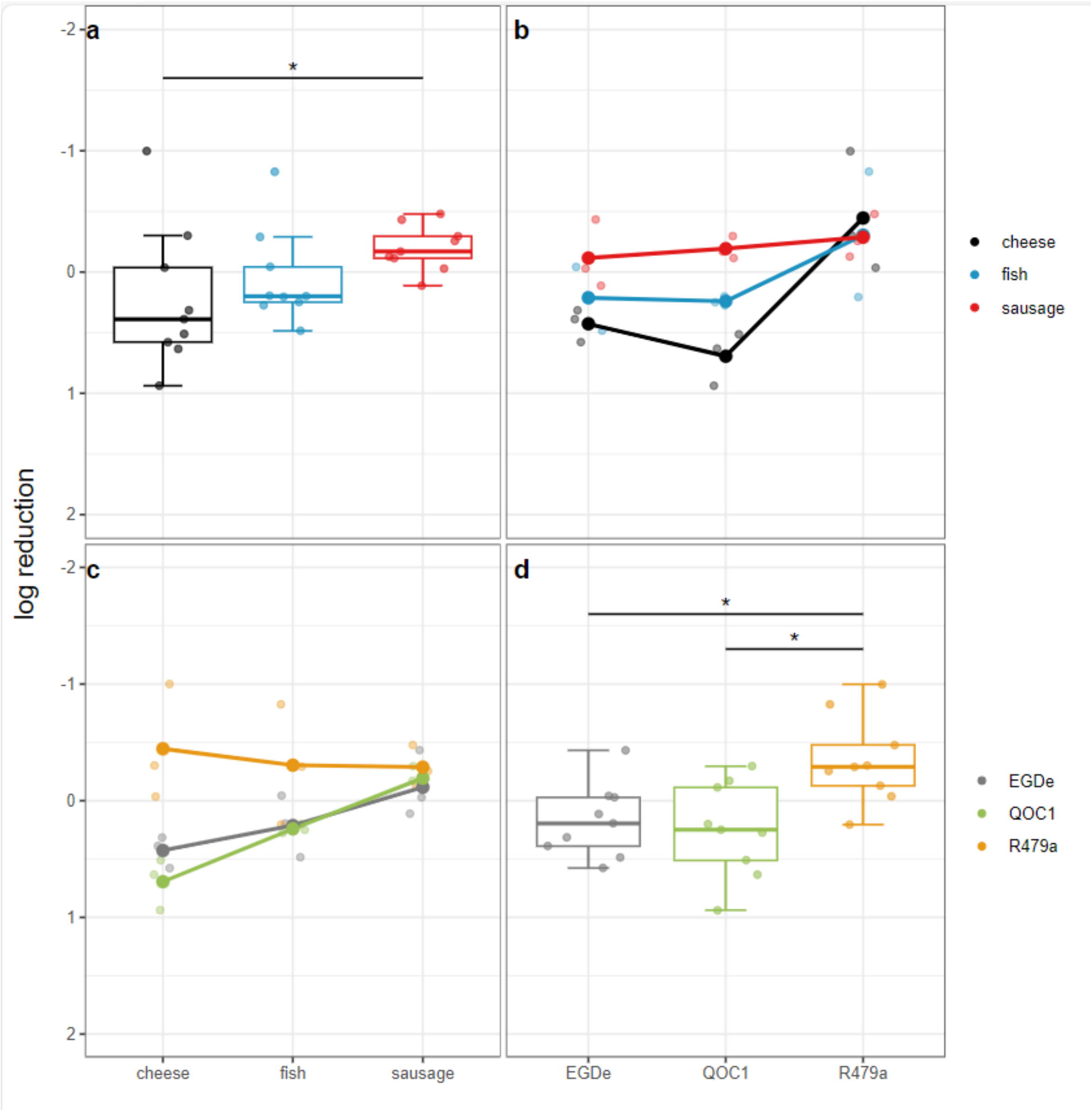

S2 Figure. Survival of *L. monocytogenes* grown for 7 days at 10 °C in different food matrices after the oral phase. Boxplot for log reduction of *L. monocytogenes* split in the different food matrices (A) and for the different strains (D). Interaction plot (log reduction) showing interactions of the different food matrices depending on the strain (B) and of the different strains depending on the food matrices (C). Black: cheese; Blue: Fish; Red: Sausage; Grey: EGDe; Green: QOC1, Orange: R479a. * $p < 0.05$

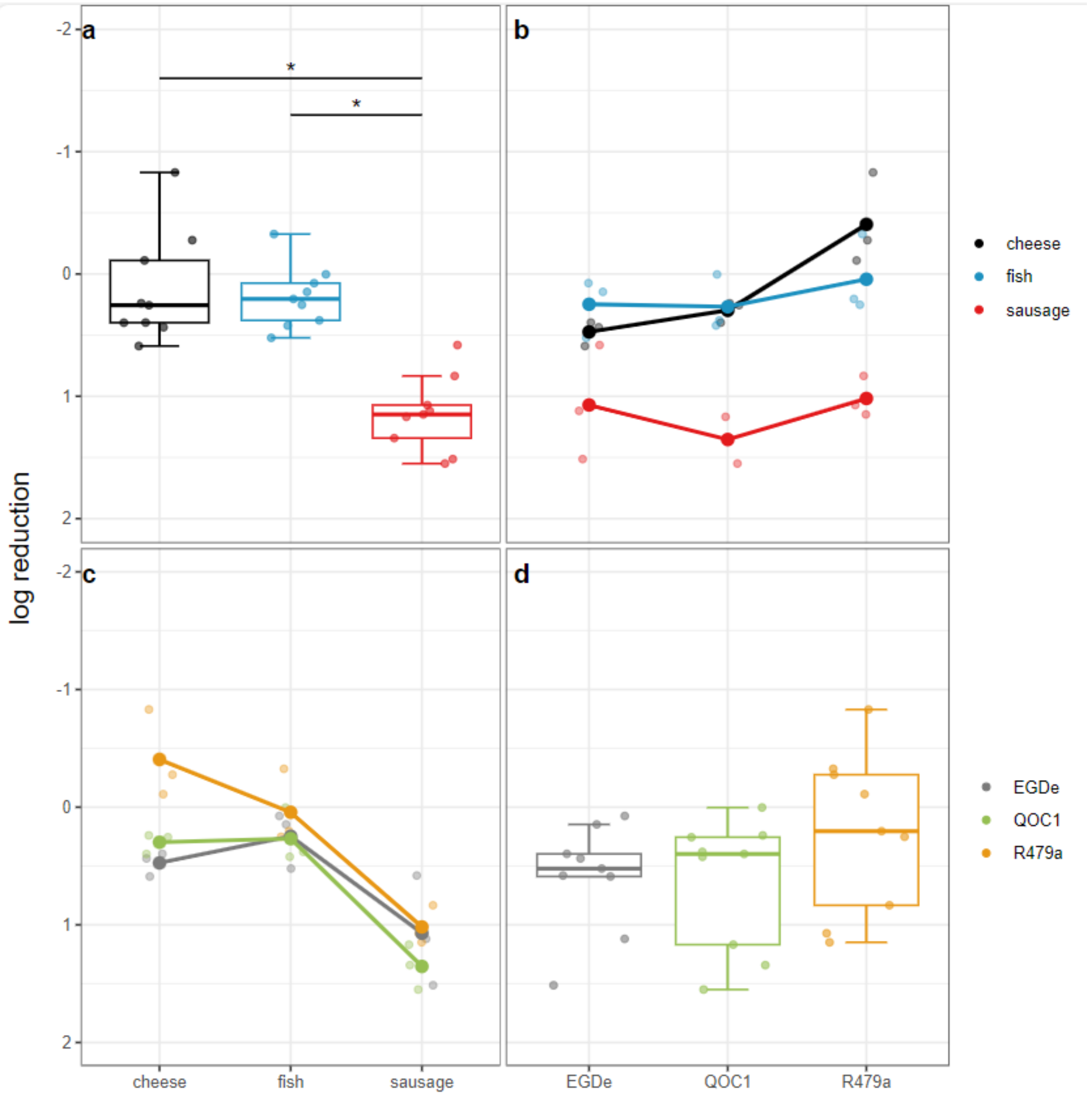

S3 Figure. Survival of *L. monocytogenes* in different food matrices after the gastric phase. Boxplot for log reduction of *L. monocytogenes* split in the different food matrices (A) and for the different strains (D). Interaction plot (log reduction) showing interactions of the different food matrices depending on the strain (B) and of the different strains depending on the food matrices (C). Black: cheese; Blue: Fish; Red: Sausage; Grey: EGDe; Green: QOC1, Orange: R479a. * $p < 0.05$

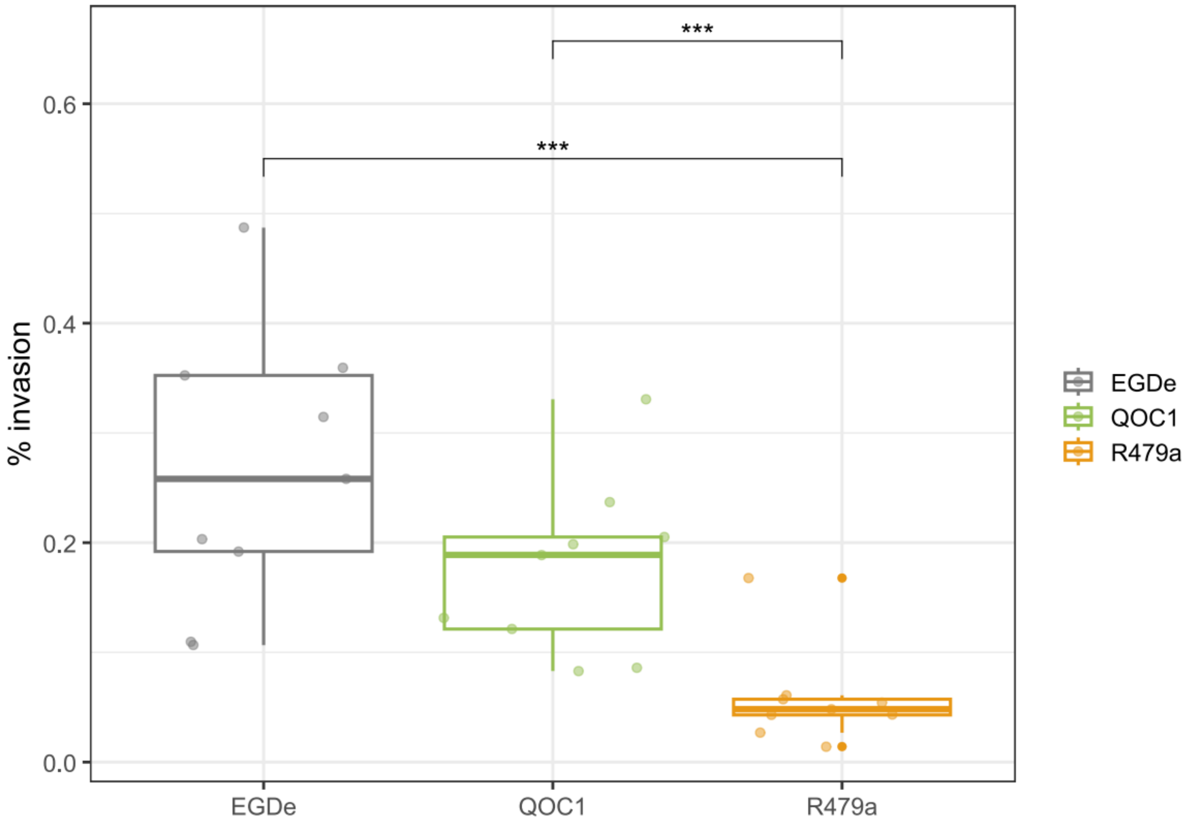

S4 Figure. Invasion of *L. monocytogenes*. Boxplot for the basal invasion efficiency (%) into Caco-2 cells without the application of previous stress conditions. Sausage; Grey: EGDe; Green: QOC1, Orange: R479a. *** $p < 0.001$

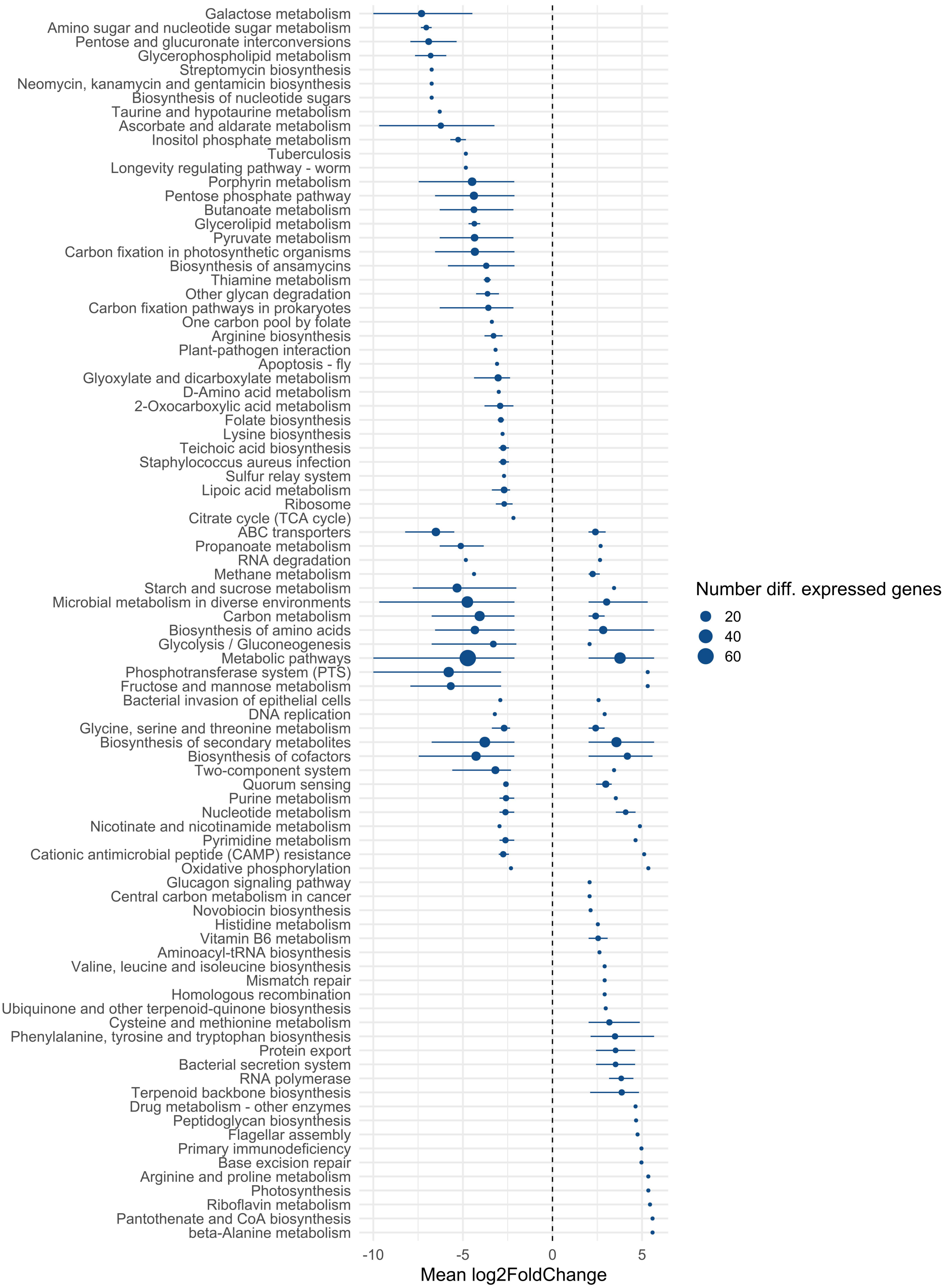

S5 Figure. Differential gene expression after seven-day growth in sausage vs. cheese. Differentially expressed genes (*L. monocytogenes* EGDe; p adj < 0.05 and log2FC ≥ |2|) were assigned to KEGG pathways. The mean log2FoldChange was calculated for downregulated or upregulated genes within each pathway, respectively. Lines indicate the range of log2FoldChange values.

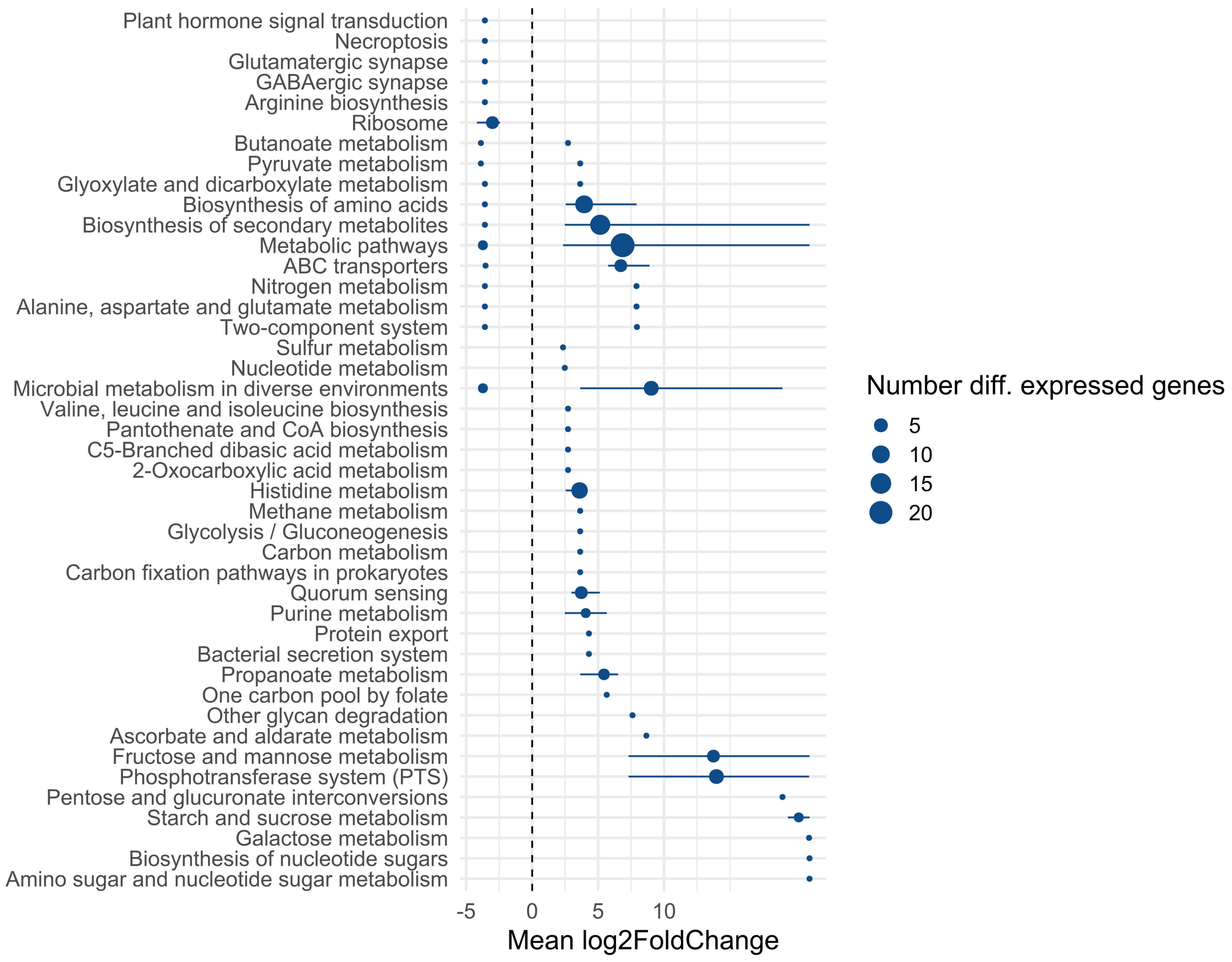

S6 Figure. Differential gene expression after seven-day growth in sausage vs. fish. Differentially expressed genes (*L. monocytogenes* EGDe; *p* adj < 0.05 and log2FC ≥ |2|) were assigned to KEGG pathways. The mean log2FoldChange was calculated for downregulated or upregulated genes within each pathway, respectively. Lines indicate the range of log2FoldChange values.

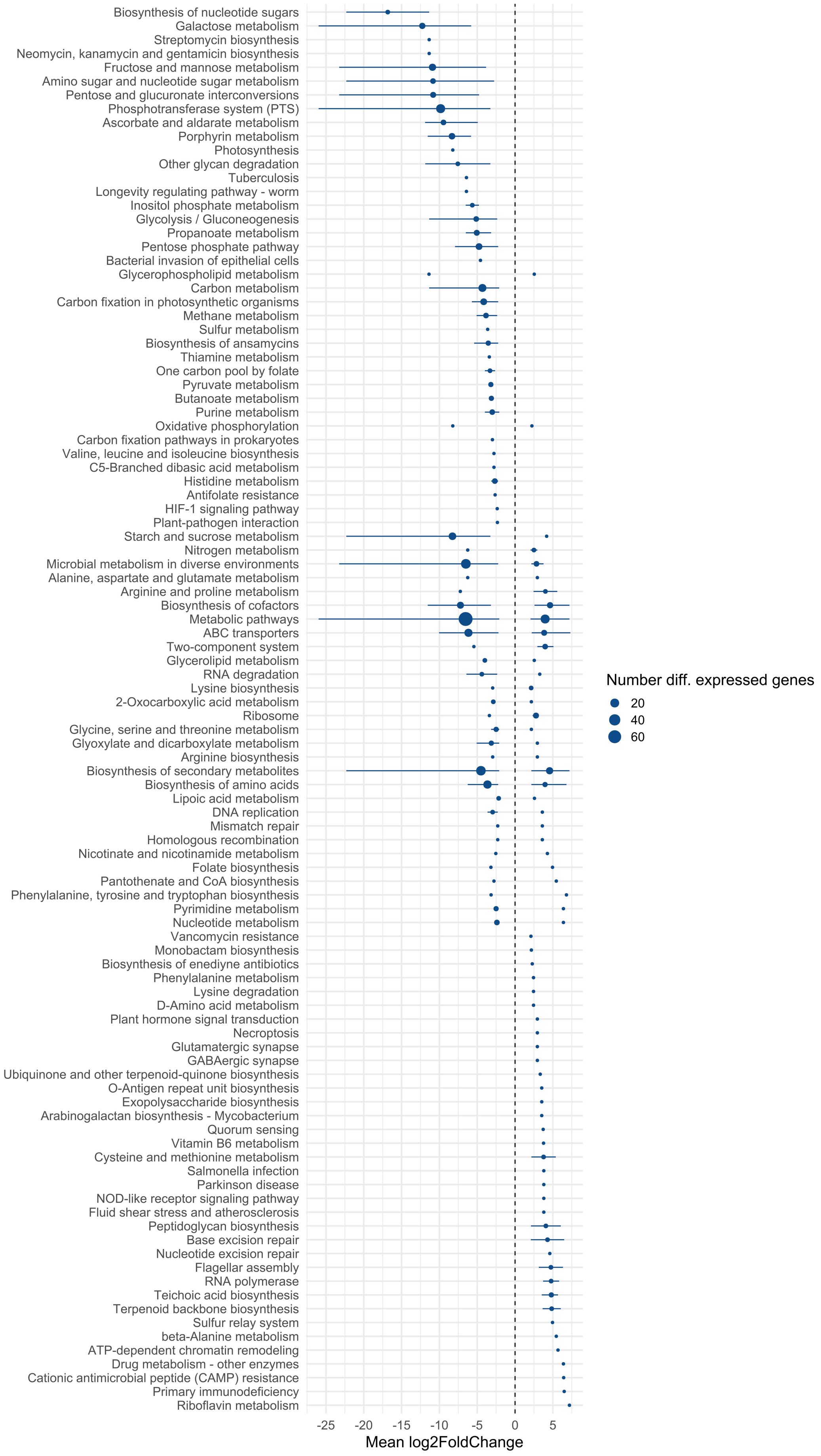

S7 Figure. Differential gene expression after seven-day growth in fish vs. cheese. Differentially expressed genes (*L. monocytogenes* EGDe; *p* adj < 0.05 and log2FC ≥ |2|) were assigned to KEGG pathways. The mean log2FoldChange was calculated for downregulated or upregulated genes within each pathway, respectively. Lines indicate the range of log2FoldChange values66.

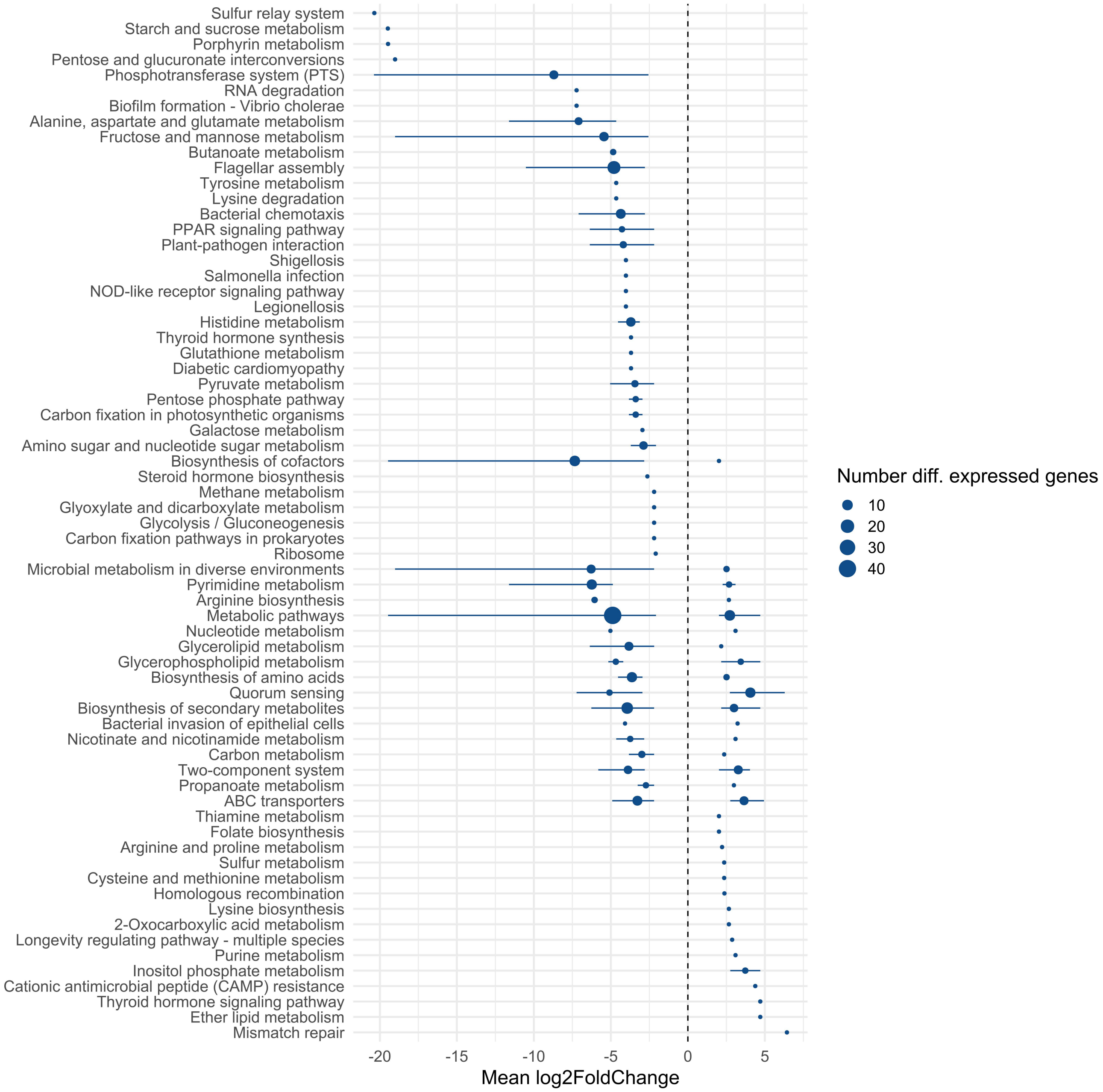

S8 Figure. Differential gene expression after passage through the GI model vs. seven-day growth in sausage. Differentially expressed genes (*L. monocytogenes* EGDe; *p* adj < 0.05 and log2FC ≥ |2|) were assigned to KEGG pathways. The mean log2FoldChange was calculated for downregulated or upregulated genes within each pathway, respectively. Lines indicate the range of log2FoldChange values.

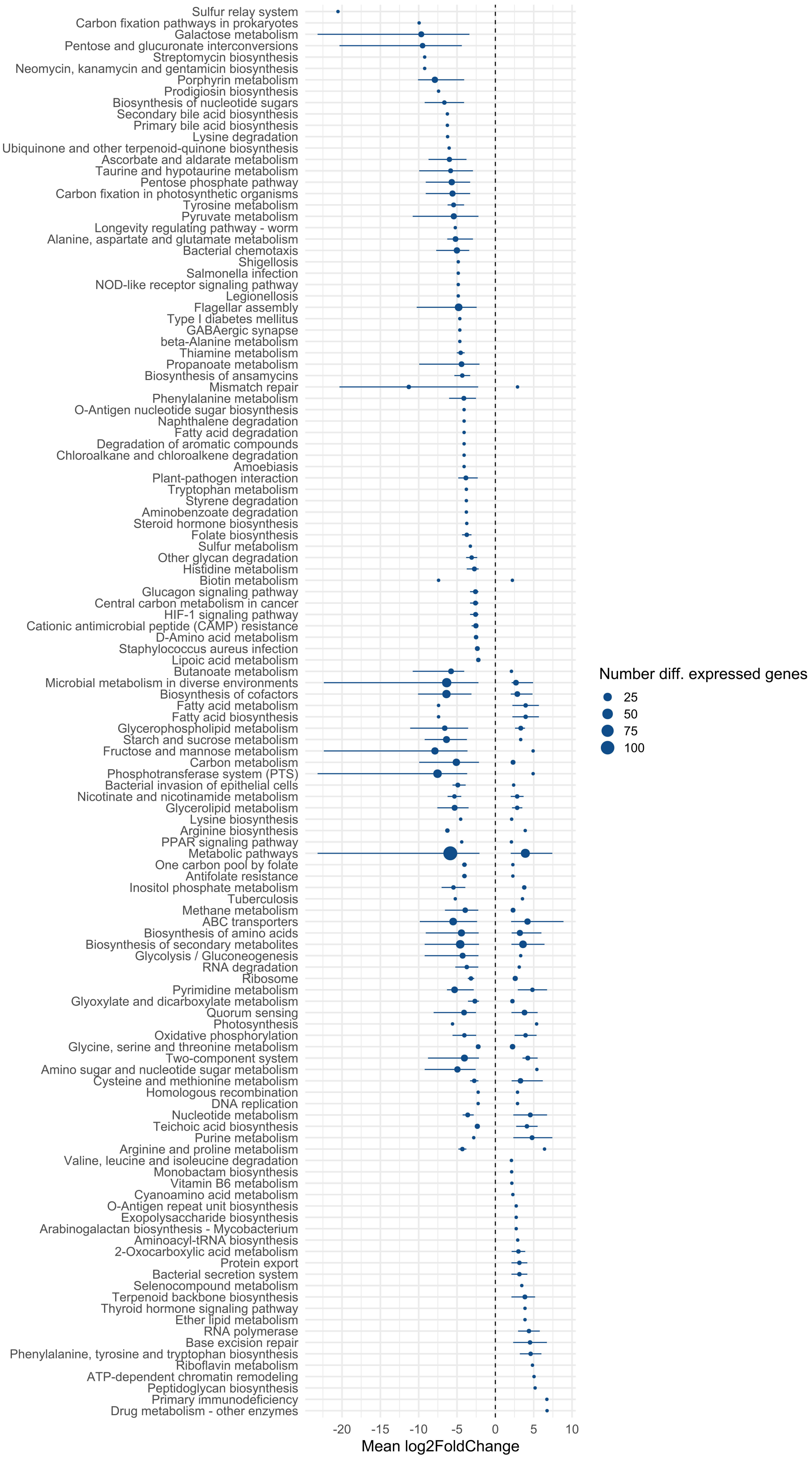

Figure legend: see next page

S9 Figure. Differential gene expression after passage through the GI model vs. seven-day growth in cheese. Differentially expressed genes (*L. monocytogenes* EGDe; *p* adj < 0.05 and log2FC ≥ |2|) were assigned to KEGG pathways. The mean log2FoldChange was calculated for downregulated or upregulated genes within each pathway, respectively. Lines indicate the range of log2FoldChange values.

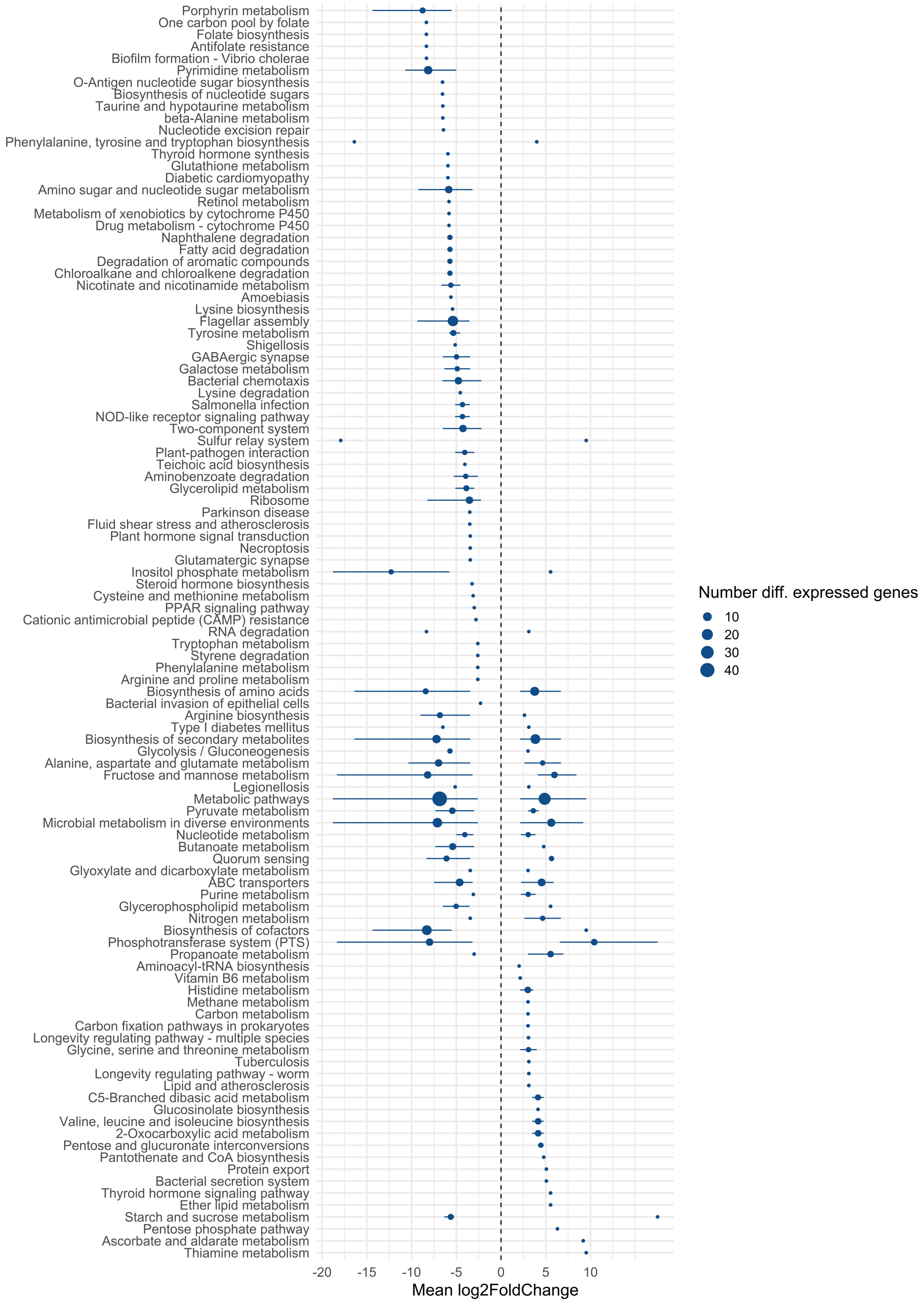

Figure legend: see next page

S10 F i g u r e . Differential gene expression after passage through the GI model vs. seven-day growth in fish. Differentially expressed genes (*L. monocytogenes* EGDe; *p* adj < 0.05 and log2FoldChange ≥ |2|) were assigned to KEGG pathways. The mean log2FoldChange was calculated for downregulated or upregulated genes within each pathway, respectively. Lines indicate the range of log2FoldChange values.

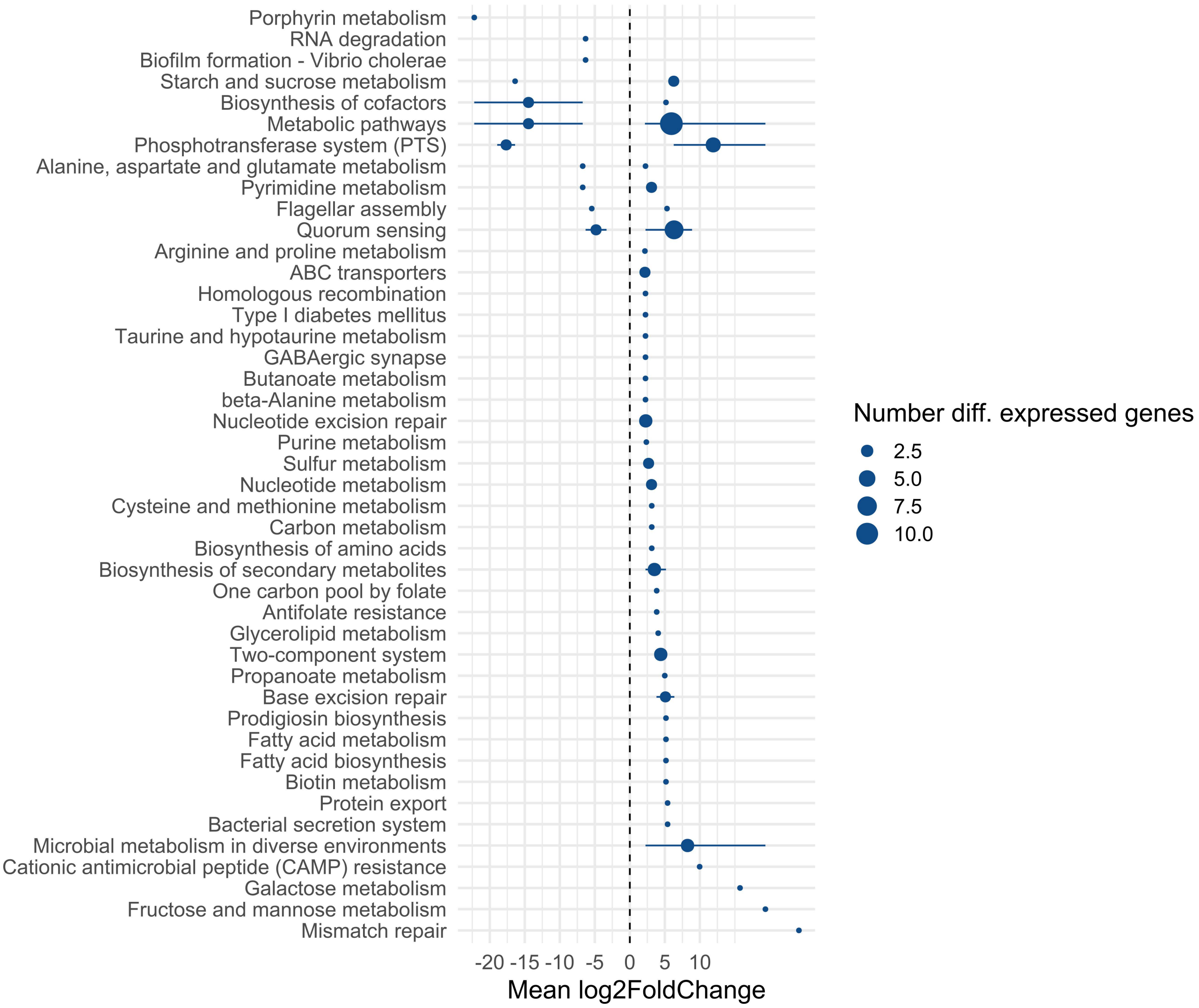

S11 Figure. Differential gene expression after passage through the GI model in sausage vs. cheese. Differentially expressed genes (*L. monocytogenes* EGDe; *p* adj < 0.05 and log2FoldChange ≥ |2|) were assigned to KEGG pathways. The mean log2FoldChange was calculated for downregulated or upregulated genes within each pathway, respectively. Lines indicate the range of log2FoldChange values.

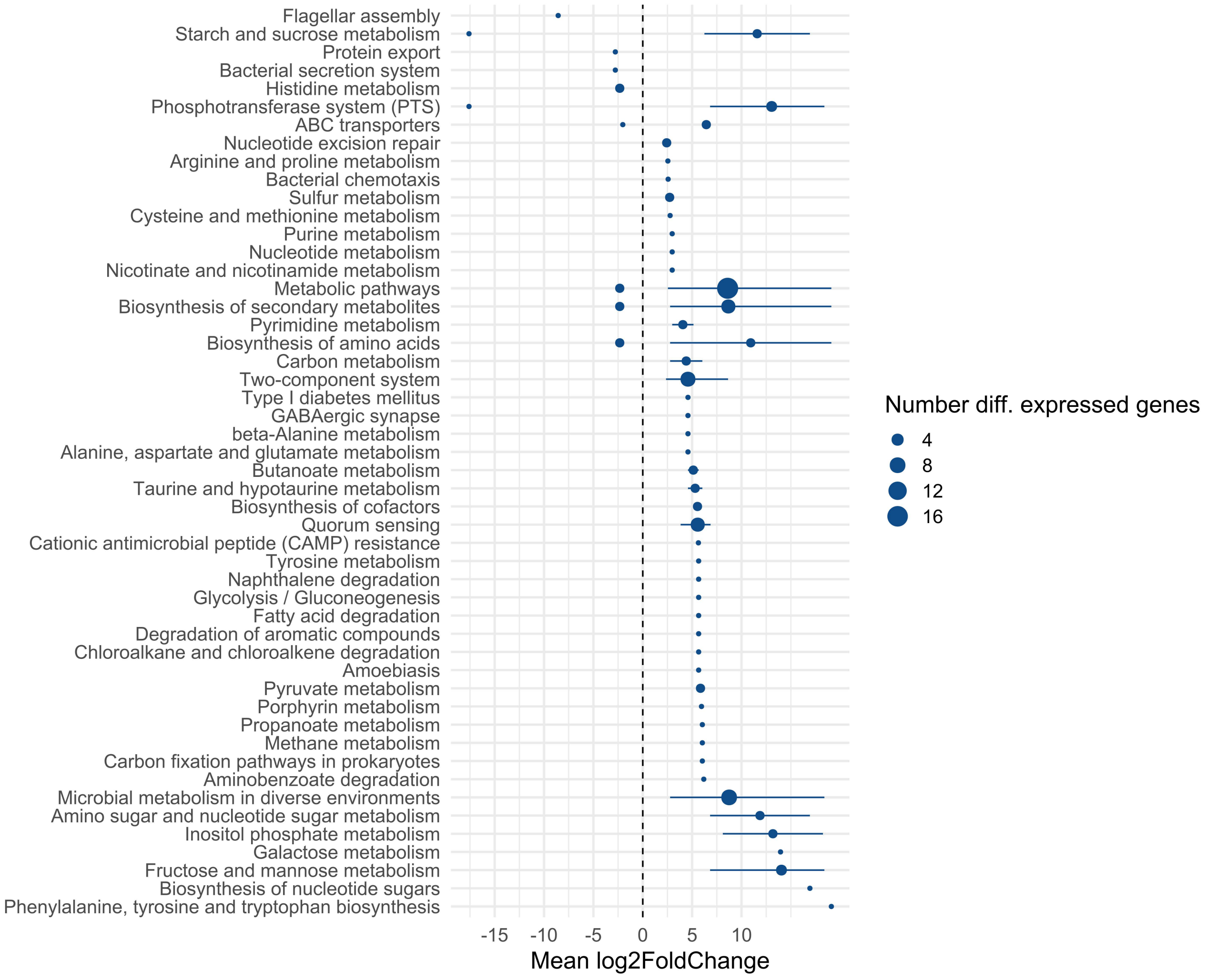

S12 Figure. Differential gene expression after passage through the GI model in sausage vs. fish. Differentially expressed genes (*L. monocytogenes* EGDe; *p* adj < 0.05 and log2FoldChange ≥ |2|) were assigned to KEGG pathways. The mean log2FoldChange was calculated for downregulated or upregulated genes within each pathway, respectively. Lines indicate the range of log2FoldChange values.

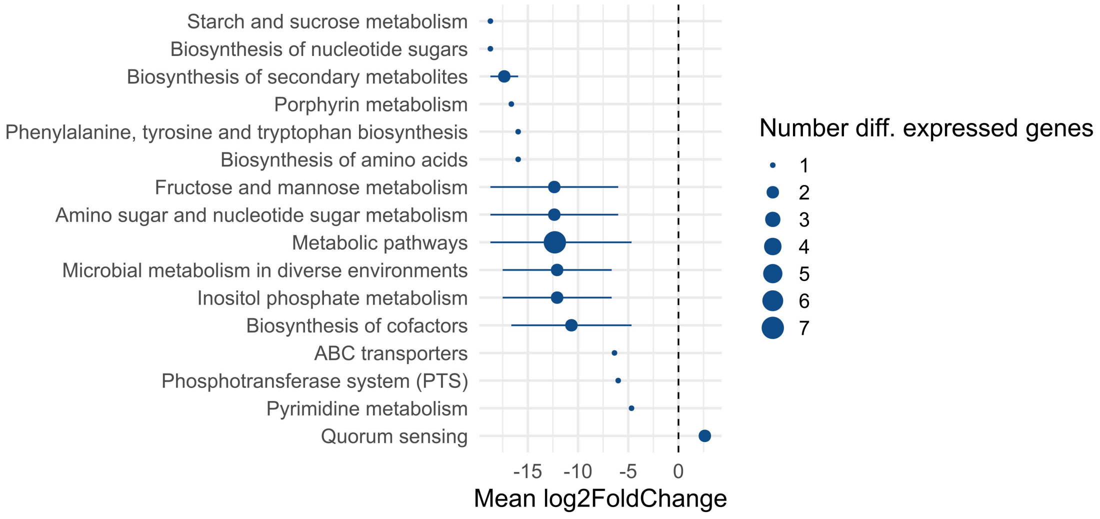

S13 Figure. Differential gene expression after passage through the GI model in fish vs. cheese. Differentially expressed genes (*L. monocytogenes* EGDe; *p* adj < 0.05 and log2FC > |2|) were assigned to KEGG pathways. The mean log2FoldChange was calculated for downregulated or upregulated genes within each pathway, respectively. Lines indicate the range of log2FoldChange values.

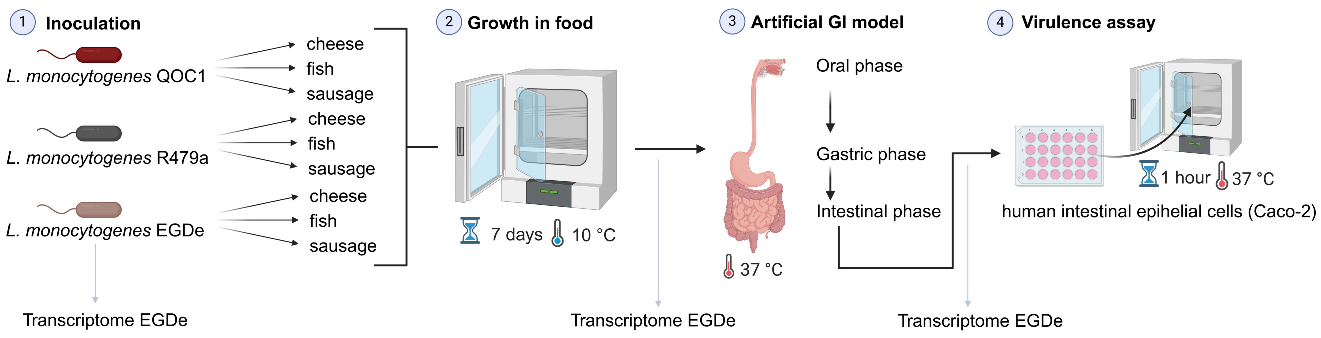

S14 Figure. Experimental set-up. Cheese, fish and sausage were inoculated with three single *L. monocytogenes* strains (EGDe, R479a, QOC1) in single experiments, respectively. The inoculated food incubated at 10 °C for seven days. Next, the inoculated food matrices were transferred to a three-steps artificial gastrointestinal (GI) model inluding oral, gastric and intestinal phases. In the last step, *L. monocytogenes* were recovered from the digested food matrices and the invasion ability was assessed using human intestinal epithelial Caco-2 cells. Colony forming units (CFU) were determined using the inocula, after growth in food, after each step of the GI model and after the virulence assay. In addition, *L. monocytogenes* RNA was isolated from the inoculum, after growth in the different types of food and after the GI model (EGDe only).
Created in BioRender. Pracser, N. (2025) https://BioRender.com/z68g488
